# Delta/Lambda Chirality: From Enantiomers to Diastereomers in Heterometallic Complexes with Chelating Ligands

**DOI:** 10.1021/acs.inorgchem.5c01670

**Published:** 2025-06-20

**Authors:** Yuxuan Zhang, Haixiang Han, Zheng Wei, Muhammad Zulqarnain, Tieyan Chang, Yu-Sheng Chen, Evgeny V. Dikarev

**Affiliations:** † Department of Chemistry, 1084 University at Albany, SUNY, Albany, New York 12222, United States; ‡ School of Material Science and Engineering, 12476 Tongji University, Shanghai 201804, China; § NSF’s ChemMatCars, Center for Advanced Radiation Source, 2462 The University of Chicago, Argonne, Illinois 60439, United States

## Abstract

The Δ/Λ chirality observed in octahedral molecules with chelating ligands represents the major group of “chiral-at-metal” complexes. Upon shifting from mononuclear to polynuclear systems with multiple (≥2) chiral centers, not only enantiomers but also diastereomers should be considered. We present the first, to the best of our knowledge, diastereomeric pairs Δ,Δ,Δ/Λ,Λ,Λ (**1**) and Δ,Δ,Λ/Λ,Λ,Δ (**2**) of the pentanuclear assembly [Mn^II^(ptac)_3_–Na-Co^III^(acac)_3_–Na-Mn^II^(ptac)_3_] (ptac = 1,1,1-trifluoro-5,5-dimethyl-2,4-hexanedionate; acac = acetylacetonate). Diastereomers **1** and **2** were isolated in pure form and found to exhibit distinctly different structural characteristics. Importantly, for compounds that are applied as single-source precursors for the quaternary oxide cathode material P2–Na_0.67_Mn_0.67_Co_0.33_O_2_, the diastereomers revealed different thermal behaviors in terms of volatility and thermal stability. Unambiguous assignment of the Mn and Co positions in both diastereomers has been confirmed by the synchrotron X-ray resonant diffraction technique. Oxidation states of metal ions have been verified by the synchrotron X-ray fluorescence spectroscopy. The *diastereomerization* between **1** and **2** is not taking place in the solid state (crystal-to-crystal), as well as in the gas phase. The transformation between two diastereomers was observed in the solutions of noncoordinating solvents and was related to the polarities of the solvents and diastereomeric molecules.

## Graphical abstract

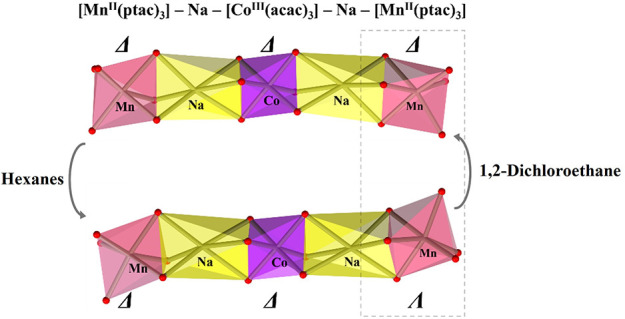

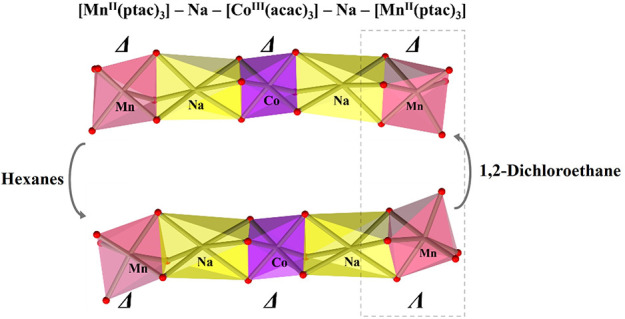

## Introduction

A molecule is considered to be chiral when it differs from its mirror image.^1^ When it comes to the chiral metal complexes,^2,3^ they can be classified as “chiral-at-ligand” or “chiral-at-metal”.^4,5^ The stereometric effect on “chiral-at-ligand” complexes, considered as a part of “ligand isomerism”,^6–8^ is typically caused by the *R*-/*S*-chiality^9^ in one of the ligands. The discussion of “chiral-at-metal” complexes mostly revolves around the coordination number six of the central metal atom.^10,11^ The systems under consideration include at least three different monodentate ligands^5^ or multidentate groups.^12^ The latter type has been studied in polynuclear complexes,^13–16^ clusters,^17,18^ and MOFs^19^ with some applications such as asymmetric catalysis.^4^

The Δ/Λ chirality observed in octahedral molecules with chelating ligands clearly represents the major group of “chiral-at-metal” complexes.^20^ The textbook example of this broad family is *tris*(bipyridine) ruthenium­(II) complex, featuring Δ and Λ-enantiomers.^21^ In general, the Δ/Λ chirality exists in the mononuclear octahedral complexes with three (Scheme 1, top) or even two (Scheme 1, bottom left) chelating ligands, no matter whether the ligands are symmetric or unsymmetric. However, in the latter case, two chelating ligands should be in the *cis-*arrangement, since the *trans*-configuration has an intramolecular mirror plane (Scheme 1, bottom right).

**Scheme 1:**
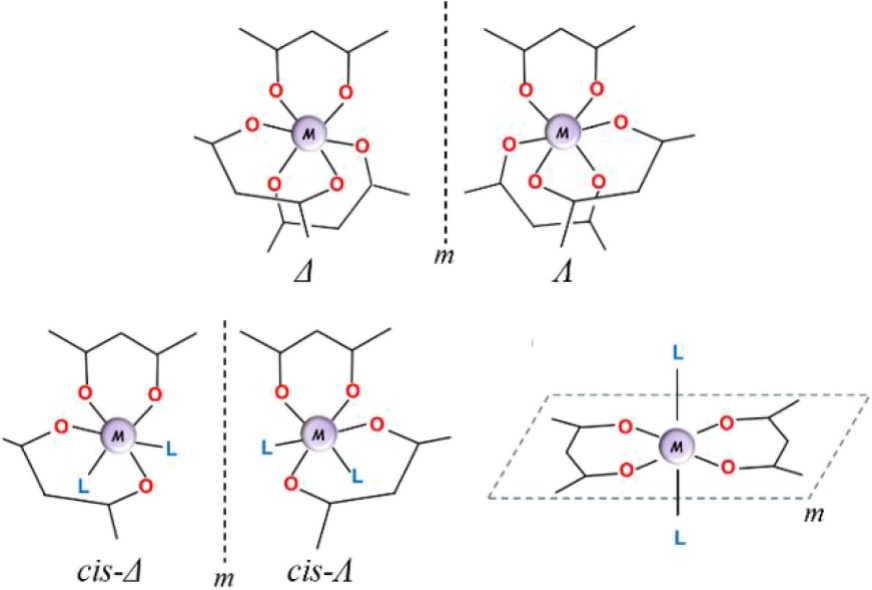
Δ/Λ Chirality in Mononuclear Octahedral Metal Complexes with Chelating Ligands

The typical chelating ligands participating in chiral mononuclear metal complexes include dicarboxylates,^22^ diamines,^23^ bipyridyls,^24^ diphosphines,^25^ and diketonates^26,27^ among others. When transitioning from mononuclear to polynuclear systems, not only enantiomers but also diastereomers^28^ (i.e., ΔΔ-/ΔΛ-) should be considered. The polynucleating ligands, defined as “a class of ligands able to simultaneously bind two or more metal ions”,^29–31^ are capable of making polynuclear systems and include primarily chelating groups such as phenolic pyrazoles,^31^ salen-type ligands,^32,33^ β-keto esters,^34^ and β-diketonates.^35,36^ Among those, a great number of polynuclear β-diketonate complexes, both homo- ^14,15^ and heterometallic,^37^ have been discovered due to the good chelating-bridging ability of this ligand type. The first polynuclear diketonate complex, [Ni_3_(acac)_6_], reported by Pauling in 1961,^13^ features *cis*-Δ and *cis-*Λ [Ni­(acac)_2_] units on both sides of the trinuclear assembly. The tetranuclear counterpart, [Co_4_(acac)_8_], published by Cotton in 1964,^15^ consists of four *cis-*[Co­(acac)_2_] units with a ΔΔΛΛ-configuration. Both structures are *meso* with no chiral forms having been detected.

Upon the continuing exploration of polynuclear heterometallic β-diketonate complexes, the structures with two,^38^ three,^38^ four,^15^ six,^39^ as well as with an infinite number of chiral centers^40^ have been characterized; still, no diastereomers were detected. Notably, most polynuclear (heterometallic) metal β-diketonate complexes are *meso*. These optically inactive compounds always result from the presence of an intramolecular inversion center, strikingly differing from organic *meso* molecules typically defined by the mirror plane. And while most of the organic *meso* compounds usually feature chiral forms, no diastereomers are known for the inorganic counterparts. In addition, a number of polynuclear/heterometallic β-diketonates and β-ketoesters have cyclic structures.^40–43^ No diastereomers that would require an opening of the cyclic arrangement were found for these molecules.

It is worth noting that the discussion of chiralities in polynuclear metal β-diketonates complexes is not limited by the octahedral geometry, but also expands to the coordination numbers of four (Li),^41^ five (Na, Zn),^43,44^ six (Bi),^45^ seven (Ln),^46^ eight (Dy),^47^ and nine (Eu),^48^ with no diastereomeric forms been reported for the corresponding molecules.

It is evident that the lack of diastereomers among polynuclear β-diketonate complexes does not mean they cannot be synthesized and isolated. The fact that diastereomers should possess different properties,^49^ including reactivity,^50^ points out that the synthetic conditions leading to diastereomeric forms should be different. In retrospect, very limited research has been devoted to the preparation of polynuclear complexes using different conditions, such as solution vs solid-state vs gas phase, or low vs high temperature, or polar vs nonpolar solvents. In addition, the characterization of the products should not only focus on the crystals, but rather on the investigation of the bulk reaction products, especially those obtained with short reaction/isolation times. The best technique for such interrogation of the solid samples is powder X-ray diffraction, which has certainly been missed in most of the previous studies. Many of the interesting polynuclear diketonate complexes basically constitute single examples with no variety of metals or ligands that have been discovered a long time ago and used simply as starting reagents since then.

Herein, we report the first, to the best of our knowledge, inorganic diastereomers discovered in heterometallic pentanuclear compounds with primarily chelating ligands that have been isolated in pure form and characterized.

### Rationale for the Diastereomer Hunt in Heterometallic Systems

In order to search for diastereomers, we have selected large families of heterometallic compounds with a certain asymmetry (not *meso*) and compositional complexity, as well as an additional degree of flexibility in their structures. Two such hetero*tri*metallic systems have been identified: trinuclear [L_3_M^II^-Na-M′^III^L′_3_] (Scheme 2, top)^51^ and pentanuclear [L_3_M^II^-Na-M′^III^L′_3_-Na-M^II^L_3_] (Scheme 2, bottom).^52^ These two families have been designed as single-source molecular precursors for the quaternary oxides with Na:M:M′ ratios of 1:1:1 and 2:2:1, respectively, which are prospective cathode materials for the sodium-ion batteries. The hetero*tri*metallic structures can incorporate a variety of divalent and trivalent metals. In addition, the octahedrally coordinated chiral transition metal centers are not bridged directly but rather through the “naked” Na ions, thus adding to the structural flexibility of the assemblies.

**Scheme 2:**
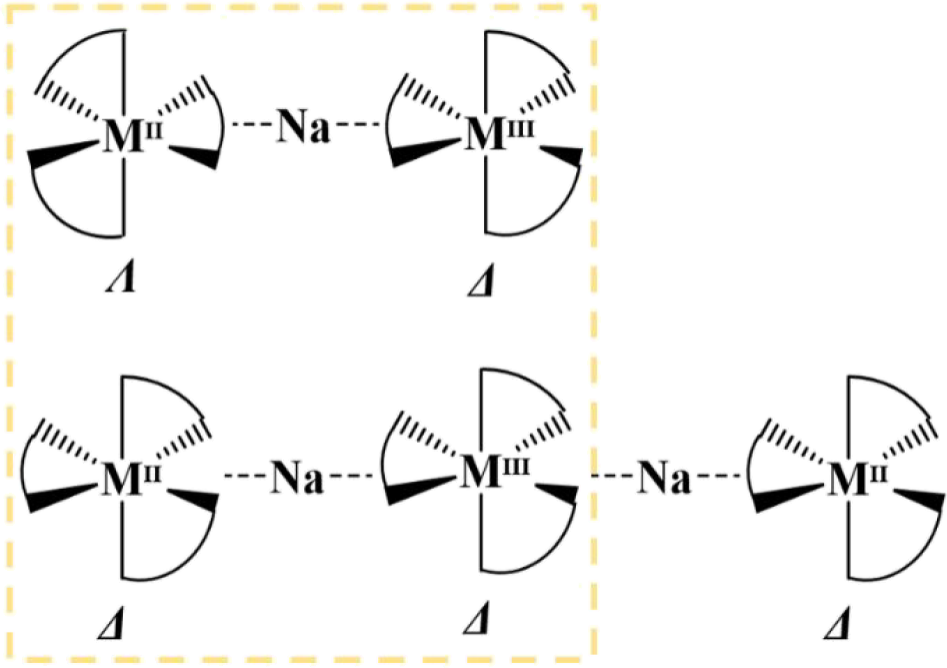
Chiralities of the Common Fragment [L_3_M^II^-Na-M^III^L_3_] in Trinuclear [M^II^(hfac)_3_-Na-M^III^(acac)_3_] (Top) and in Pentanuclear [M^II^(ptac)_3_-Na-M^III^(acac)_3_-Na-M^II^(ptac)_3_] (Bottom) Molecules

Importantly, the molecules of these two families feature a common fragment, [L_3_M^II^-Na-M^III^L_3_], in their structures (Scheme 2).^51^ Interestingly, from the analysis of the structures reported so far, the chiralities of the above elements appear to be different: Λ,Δ (Δ,Λ) in the trinuclear assembly and Δ,Δ (Λ,Λ) in pentanuclear assemblies (Scheme 2). The latter basically implies that the title “fragment” can exist as diastereomeric, since it is not affected by the steric constraints that may prevent the appearance of different configurations in these molecules. The trinuclear assembly was initially selected for a careful investigation. These molecules potentially have two diastereomeric pairs, Λ,Δ/Δ,Λ and Δ,Δ*/*Λ,Λ. However, despite the successful preparation of [L_3_M^II^-Na-M^III^L_3_] molecules with nearly all possible combinations of divalent and trivalent ions (hetero*bi*- and *tri*metallic, main group and transition metals, and large and small ions), none of the diastereomers have been detected as of now. An attestation of our meticulous search is the discovery of ionic and molecular isomers^53^ that represent the first, to the best of our knowledge, example of such type of isomerism in polynuclear coordination compounds.

Therefore, we turned our attention to the family of pentanuclear molecules that might have even more structural flexibility compared with their trinuclear counterparts. These molecules featuring three *tris*-chelated chiral metal centers may theoretically appear as three diastereomeric pairs (Scheme 3). While only three members of these pentanuclear molecules have been reported,^52^ our synthetic study reveals that a number of divalent and trivalent metals can easily fit into this assembly. That said, all pentanuclear molecules [L_3_M^II^-Na-M^III^L_3_-Na-M^II^L_3_] isolated so far are known as the Δ,Δ,Δ*/*Λ,Λ,Λ enantiomeric pair. We started to carefully investigate the powder X-ray diffraction patterns of quickly isolated bulk reaction products obtained in different solvents at different temperatures using a variety of starting reagents in order to compare the data with those of known Δ,Δ,Δ*/*Λ,Λ,Λ complexes.

**Scheme 3:**
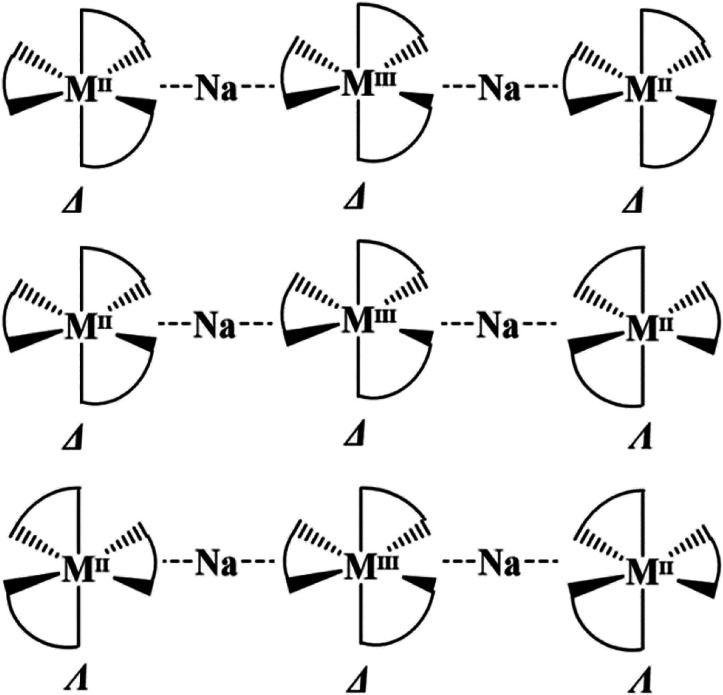
Three Possible Diastereomers of Pentanuclear Molecule [M^II^(ptac)_3_-Na-M^III^(acac)_3_-Na-M^II^(ptac)_3_]

## Results and Discussion

### Synthesis of Heterometallic Isomers **1** and **2**

Carrying out reaction (eq 1) in moist and oxygen-free hexanes by stirring at room temperature for 12 h, green-colored crystals of different shapes (plates and prisms) were found to crystallize out from the solution at −20 °C within 3 days. The ICP-OES analysis of the crystals showed the metal ratio of Na:Mn:Co as 2:2:1, corresponding to that in the pentanuclear assembly as well as to the stoichiometric process (eq 1). However, the X-ray powder diffraction pattern of the crystals, while showing that the major component in the mixture is similar to the known^52^ ΔΔΔ/ΛΛΛ-[Mn^II^(ptac)_3_NaFe^III^(acac)_3_NaMn^II^(ptac)_3_] (Figure 1), revealed that the product is not pure, despite the correct metal ratio for the bulk sample.

$$6\mathrm{Na}(\mathrm{ptac})+2{\mathrm{MnCl}}_{2}+[\mathrm{Co}(\mathrm{acac}{)}_{3}]\to [{\mathrm{Na}}_{2}{\mathrm{Mn}}_{2}\mathrm{Co}(\mathrm{acac}{)}_{3}(\mathrm{ptac}{)}_{6}]+4\mathrm{NaCl}$$ 1

**Figure 1:**
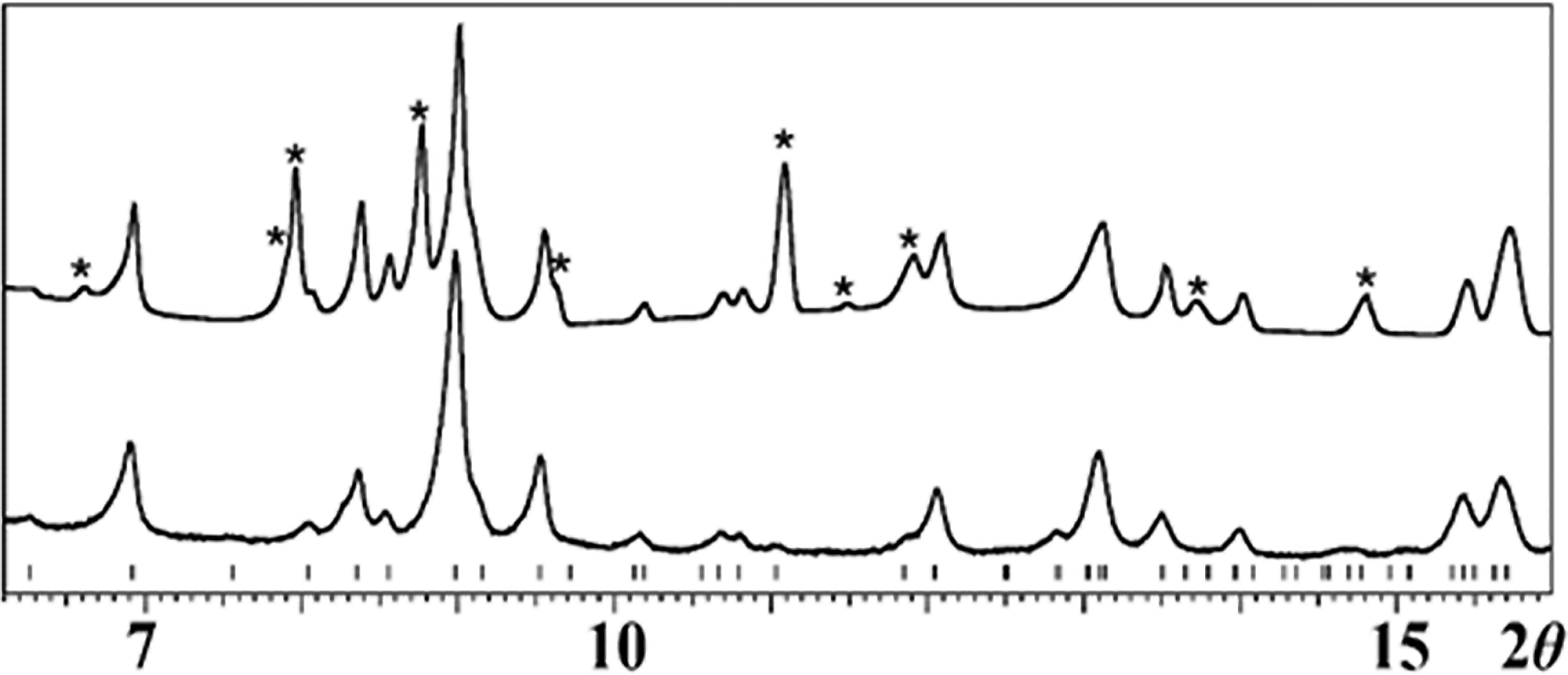
Powder X-ray diffraction patterns of the crystals obtained from the reaction (1) in hexanes (top) and the simulated pattern of the ΔΔΔ/ΛΛΛ-[Na_2_Mn_2_Fe­(acac)_3_(ptac)_6_][Bibr ref52] (bottom). Theoretical peak positions for the latter are marked with black bars at the bottom. Peaks belonging to the second phase are denoted with asterisks.

Analysis of the powder X-ray diffraction pattern (Figure 1) and ICP-OES results for the products of reaction (eq 1) points out the presence of two isomers with the Na:Mn:Co ratio of 2:2:1. However, this reaction is far from ideal because of the very low solubility of MnCl_2_ in hexanes. Substantial scale-up as well as nearly quantitative yields of the pentanuclear product have been achieved by using the interaction between stoichiometric amounts of [Co­(acac)_3_] and [NaMn­(ptac)_3_] (see the Supporting Information page S4 for detailed synthetic procedures) as starting reagents in noncoordinating organic solvents (eq 2). The isomer **1** (isomorphous to that reported^52^ for ΔΔΔ/ΛΛΛ-[Mn^II^(ptac)_3_NaFe^III^(acac)_3_NaMn^II^(ptac)_3_]) can be obtained by carrying out the reaction (eq 2) in dry, oxygen-free 1,2-dichloroethane upon 24 h stirring at room temperature. In turn, the isomer **2** can be isolated from dry, deoxygenated hexanes after 12 h of stirring at room temperature. The phase purity of two isomers was confirmed by powder X-ray diffraction (Figure 2), and the Le Bail fit was performed to show that the experimental powder patterns of **1** and **2** correspond to the theoretical ones (*vide infra*) calculated from the single crystal data (Supporting Information, Figures S1 and S2 and Tables S1 and S2).

**Figure 2:**
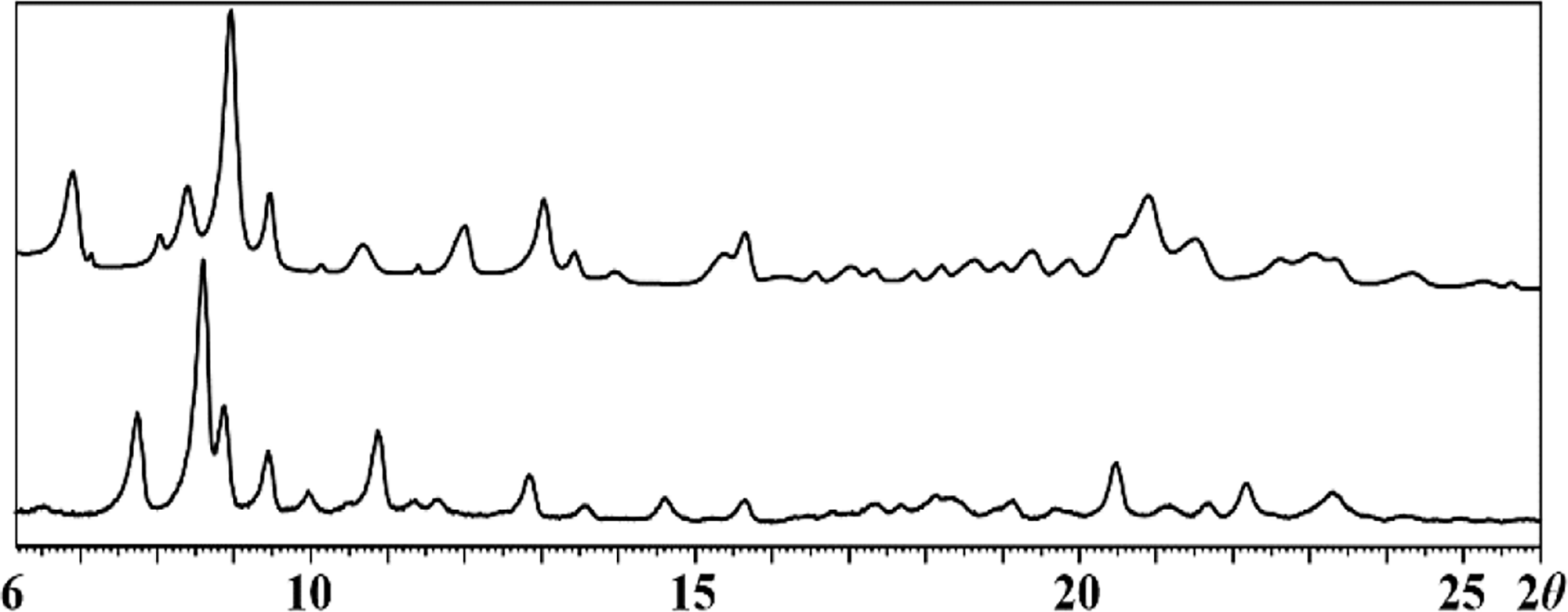
Powder X-ray diffraction patterns of isomers **1** (top) and **2** (bottom) obtained by reaction (2) carried out in 1,2-dichloethane and hexanes solutions, respectively.

$$2[\mathrm{NaMn}(\mathrm{ptac}{)}_{3}]+[\mathrm{Co}(\mathrm{acac}{)}_{3}]\to [{\mathrm{Na}}_{2}{\mathrm{Mn}}_{2}\mathrm{Co}(\mathrm{acac}{)}_{3}(\mathrm{ptac}{)}_{6}]$$ 2

### Single Crystal X-ray Structural Analysis of **1** and **2**

In-house X-ray structural analysis of **1** and **2** single crystals clearly confirmed that these two complexes with the Na:Mn:Co = 2:2:1 ratio are indeed: (1) isomers of the composition [Na_2_Mn_2_Co­(acac)_3_(ptac)_6_] as they possess the same 1:2 ratio of acac to ptac ligands and (2) diastereomers featuring Δ,Δ,Δ/Λ,Λ,Λ and Δ,Δ,Λ/Λ,Λ,Δ diastereomeric pairs, respectively. Refinement of crystal data confirmed that both **1** and **2** consist of the pentanuclear molecules [M1­(ptac)_3_-Na-M2­(acac)_3_-Na-M3­(ptac)_3_] (Scheme 4). All three transition metals (M1, M2, and M3) in each molecule are *tris*-chelated by diketonate ligands, with [M­(acac)_3_] being in the middle, and two [M­(ptac)_3_] parts located at both termini of the pentanuclear assembly. These octahedral units are bridged by two “naked” sodium centers that exhibit a distorted octahedral coordination of diketonate oxygen atoms that chelate transition metal ions. In [M­(ptac)_3_] units, only oxygen atoms located beneath the small CF_3_ substituents participate in coordination to Na ions, while the bulky *^t^* Bu groups face outward of the heterometallic assembly on both sides of the molecule, thus effectively blocking the chain growth. Hetero*tri*metallic compounds **1** and **2** are crystallized in centrosymmetric space groups (*P-*1 and *P*2_1_/*c*, respectively) with the inversion center located at the middle of the unit cell, thus making the entire pentanuclear molecule a crystallographically independent unit for both. Two diastereomers appear as a pair of enantiomers with **1** featuring Δ-[M1­(ptac)_3_], Δ-[M2­(acac)_3_], Δ-[M3­(ptac)_3_] (Scheme 4a) and Λ,Λ,Λ-enantiomers in the unit cell. Diastereomer **2** contains a pair of Δ-[M1­(ptac)_3_], Δ-[M2­(acac)_3_], Λ-[M3­(ptac)_3_] (Scheme 4b), and Λ,Λ,Δ-enantiomers in the structure. The shapes of two diastereomeric assemblies are clearly different, with **1** adopting a part of spiral configuration, if continued (Scheme 4c), while **2** assumes an essentially zigzag shape (Scheme 4d), save the chirality at the central M2 unit. Importantly, while both diastereomeric molecules do not contain an inversion center, **2** certainly appears as less polar than **1**.

**Scheme 4:**
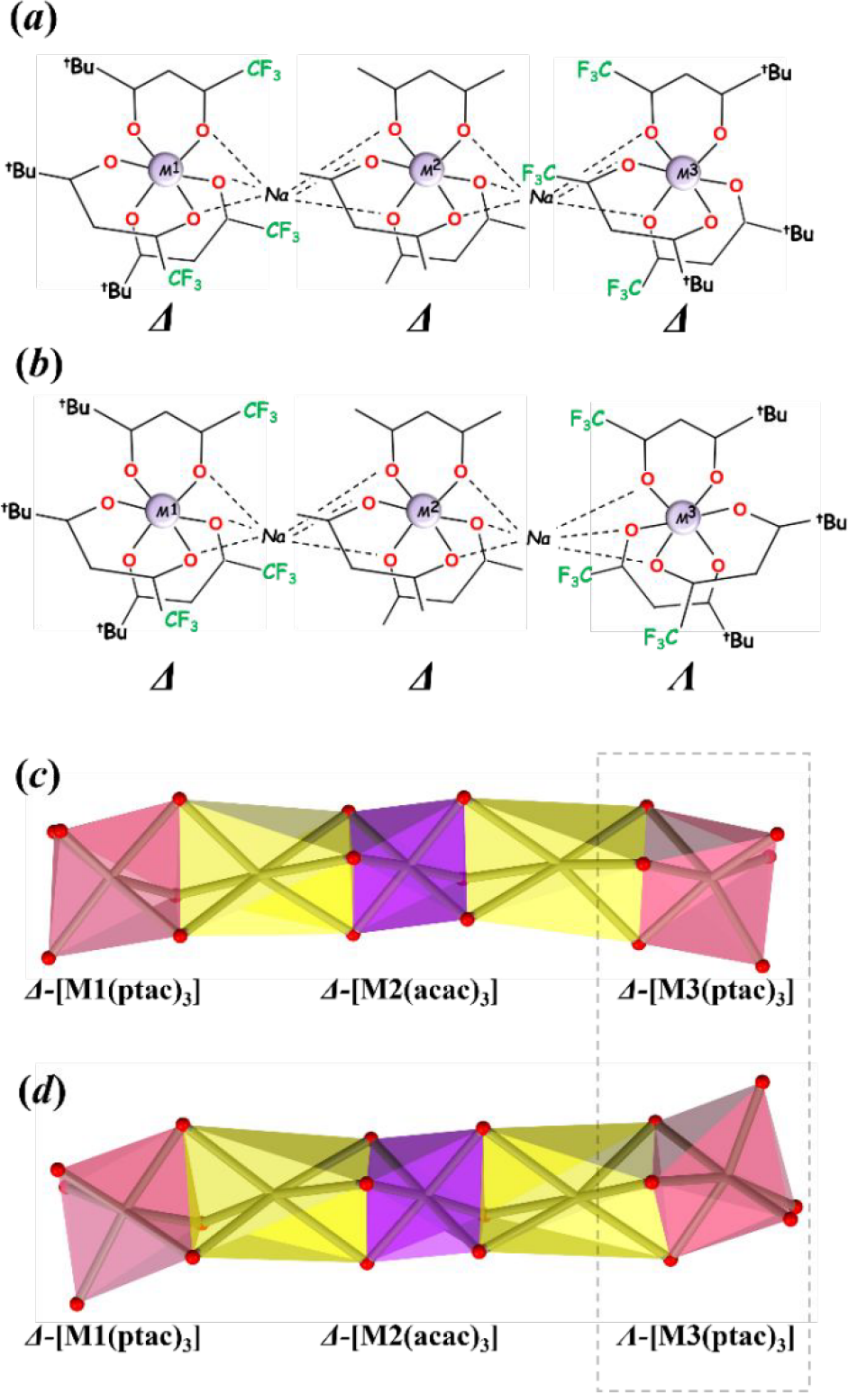
Configurations of Three *tris-*Chelated [ML_3_] Units in (a) Diastereomer **1** and (b) Diastereomer **2** ^a^

a The enantiomeric counterparts for both molecules are not shown. The polyhedral representation of the shapes of pentanuclear assemblies of (c) **1** and (d) **2**, shown as face-sharing [MO_6_] octahedral units (pink for [M­(ptac)_3_], purple for [M­(acac)_3_], and yellow for [NaO_6_]).

The use of single-crystal X-ray diffraction data for heterometallic Co–Mn compounds in order to determine the exact positions of metal ions appears somewhat problematic due to Mn and Co having close atomic numbers, masses, and radii^54^ (coordination number 6). Structural investigation of diastereomers **1** and **2** using in-house X-ray data resulted in the best convergence for the [Mn­(ptac)_3_–Na-Co­(acac)_3_–Na-Mn­(ptac)_3_] arrangement in both cases with acac groups attached to the central Co ion and ptac ligands chelating Mn ions (Supporting Information, Tables S4 and S5). Refining the structures with different Co and Mn assignments (namely, Mn–Mn–Co or Co–Mn–Mn) leads to poorer metrics, while in both cases, the refinement parameters for mixed-occupancy (Co_0.33_/Mn_0.67_ in each position) setting are quite comparable to the former assignment.

The assignment of metal positions and oxidation states in diastereomers **1** and **2** has also been attempted by analyzing the M–O bond distances in octahedral *tris*-chelated [M­(β-dik)_3_] units (Table 1). It should be noted that the electroneutrality of the pentanuclear assembly requires both structures to contain a mixed-valent (+2/+2/+3) combination of Co and Mn ions. Both diastereomers **1** and **2** exhibit very close M–O bond distances in the [M­(acac)_3_] central units and in the [M­(ptac)_3_] side units, indicating that they should have the same assignment of metals for the M1, M2, and M3 sites. It should also be stressed that the average M–O distances in the [M­(acac)_3_] and [M­(ptac)_3_] units (1.89 vs 2.14 Å, respectively) are way different in **1** and **2**, by more than it could be simply explained by the difference in ionic radii of isovalent Co and Mn ions. The latter points out different oxidation states of metals (+3 and +2) in *tris*-chelated [M^III^(acac)_3_] and [M^II^(ptac)_3_] units, respectively. Such an arrangement is in line with the previous observations^51,52^ that the ligands with electron-donating groups (acac in this case) prefer to chelate electron-poor M^III^ ions, while those with electron-withdrawing substituents (ptac) tend to coordinate relatively electron-rich M^II^ centers.

**Table 1:** Comparison of the Averaged M–O Bond Distances in Diastereomers **1** and **2** with those in the Corresponding Co and Mn [M­(β-dik)_3_] (β-dik = acac and ptac) Units

	[ref.]	M–O_acac_ (Å)	M–O_ptac_ (Å)
**1**	this work	1.8868(13)	2.1466(14)
**2**	this work	1.8815(10)	2.1358(10)
[Mn^II^(ptac)_3_NaMn^III^ (acac)_3_NaMn^II^(ptac)_3_]	^52^	1.961(5) × 2, 2.021(5) × 4	2.151(3)
[Co^II^(ptac)_3_NaCo^III^ (acac)_3_NaCo^II^(ptac)_3_]	this work^a^	1.8838(12)	2.0573(13)
[Co^III^(acac)_3_]	^55^	1.860(2)	
[Co^II^(acac)_3_]^−^	^56^	2.110(2)	
[Mn^III^(acac)_3_]	^57^	1.935(2) × 4, 2.110(2) × 2	
[Mn^II^(acac)_3_]^−^	^58^	2.171(2)	
[Mn^II^(ptac)_3_]^−^	this work^a^		2.160(2)

a Crystallographic data for [Co^II^(ptac)_3_NaCo^III^(acac)_3_NaCo^II^(ptac)_3_] and for [NaMn^II^(ptac)_3_] can be found in the Supporting Information, pages S20–S26.

The metal–oxygen distances in **1** and **2** can be directly compared with those in the parent hetero*bi*metallic counterparts, [Na_2_Mn_3_(acac)_3_(ptac)_6_] and [Na_2_Co_3_(acac)_3_(ptac)_6_]. The M–O bond distances in the [M­(acac)_3_] units of **1** and **2** are the same as in the central [Co^III^(acac)_3_] in the [Na_2_Co_3_(acac)_3_(ptac)_6_] structure, while they are very different from [Mn^III^(acac)_3_] fragments (Table 1). On the other hand, the M–O bonds in [M­(ptac)_3_] units are similar to those in [Mn^II^(ptac)_3_]^−^ in [Na_2_Mn_3_(acac)_3_(ptac)_6_] structure (Table 1), indicating the formula of two diastereomers as [Mn^II^(ptac)_3_–Na-Co^III^(acac)_3_–Na-Mn^II^(ptac)_3_] (Figure 3). In addition, none of the [ML_3_] units exhibits a Jahn–Teller effect characteristic of Mn^III^ ions known in such fragments,^52^ ruling out the presence of Mn^III^ ions in molecules **1** and **2**.

**Figure 3:**
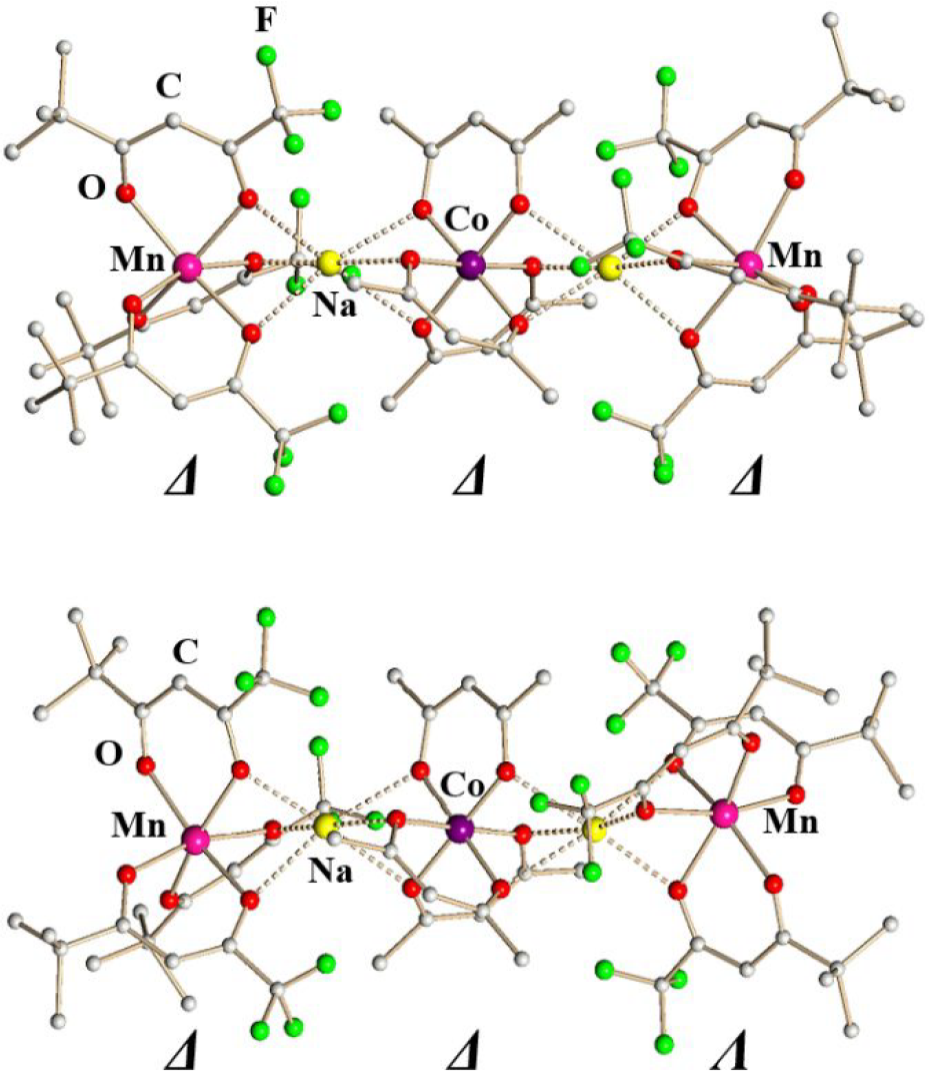
Pentanuclear units in the solid-state structures of ΔΔΔ-[Mn­(ptac)_3_NaCo­(acac)_3_NaMn­(ptac)_3_] (**1**, top) and ΔΔΛ-[Mn­(ptac)_3_NaCo­(acac)_3_NaMn­(ptac)_3_] (**2**, bottom). The bridging Na–O bonds are marked as dotted lines. All hydrogen atoms have been omitted for the sake of clarity. Full views of both structures with thermal ellipsoids and the full list of bond distances and angles are included in the Supporting Information, Figures S3 and S4 and Tables S6 and S7.

### Unambiguous Assignment of the Metal Positions and Oxidation States of Co and Mn Ions in Diastereomers **1** and **2**

In order to precisely determine each metal site occupancy factor, synchrotron X-ray resonant single crystal diffraction investigations were carried out for both diastereomers **1** and **2**, utilizing the advantages of the sensitive *K-*edge absorption at the characteristic wavelengths. This method has been demonstrated^51,52,59^ to successfully distinguish the Periodic Table neighbors based on significant differences in the anomalous dispersion factors of the elements around their absorption edges. A total of five data sets at different wavelengths (two near the Mn *K*-edge, two near the Co *K*-edge, and one away from the above absorption edges, i.e., 30 keV) were collected using a synchrotron radiation source. The structural models derived from the 30 keV data were refined against those data sets near both *K*-edges to inspect the composition of transition metal positions in the [M­(acac)_3_] and [M­(ptac)_3_] units. Analysis of the anomalous difference Fourier electron density maps provides visual pictures of the metal site occupation patterns. Data sets measured at the wavelengths near the *K*-edges (Figure 4) show deep electron density holes for the respective crystallographically independent metal positions thus revealing the presence of only Co in the [M­(acac)_3_] units (Figure 4a,b) and only Mn in the [M­(ptac)_3_] fragments (Figure 4c,d) for both diastereomeric structures. The corresponding site occupancy factor refinements give the ratios of Mn:Co:Mn as 0.984(4):0.989(4):0.990(4) and 1.040(8):1.010(7):1.020(8) in **1** and **2**, respectively (see the Supporting Information, page S10 for detailed experimental procedures).

**Figure 4:**
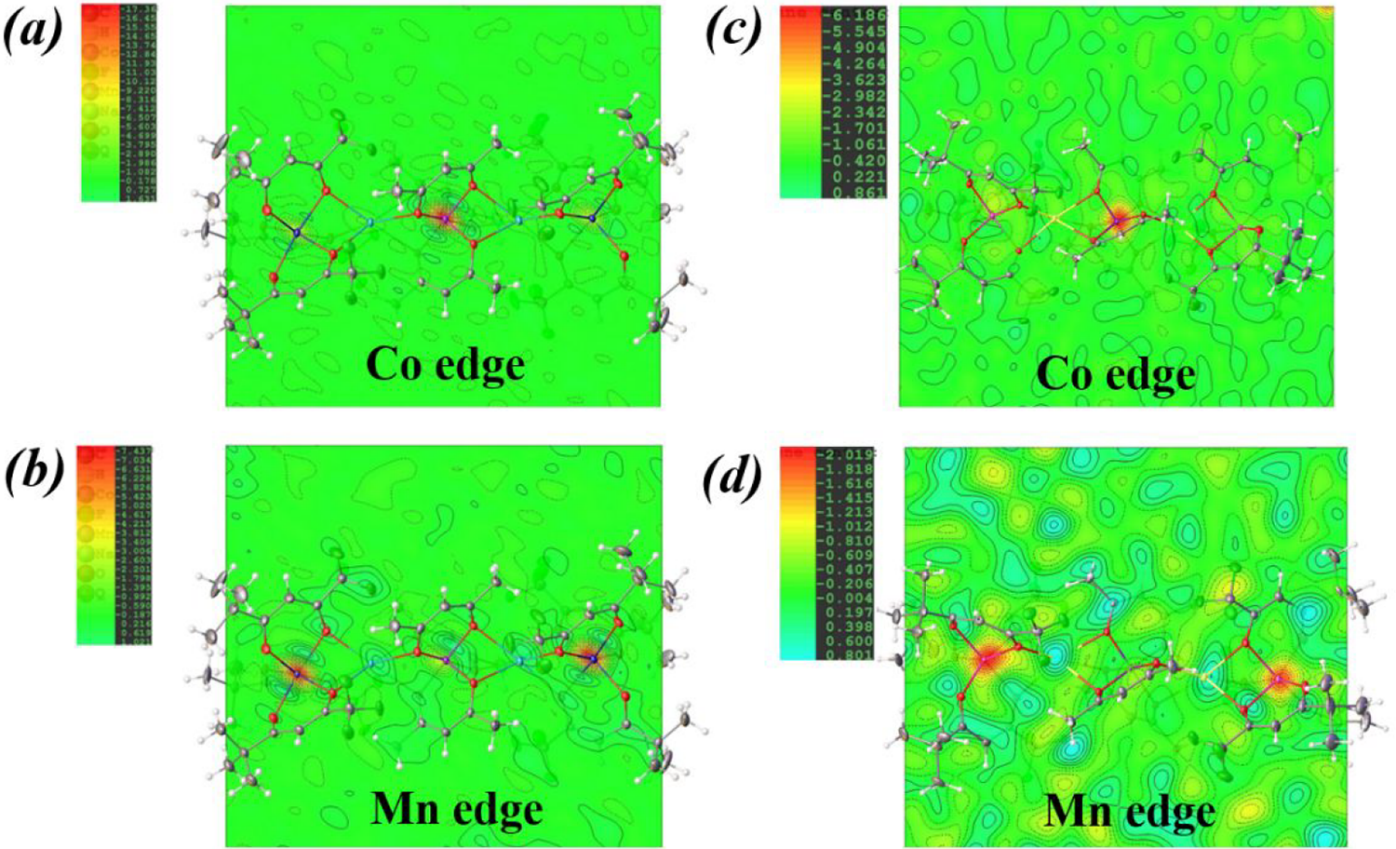
Difference Fourier electron density maps at absorption *K-*edges of (a) Co in **1**; (b) Mn in **1**; (c) Co in **2**; and (d) Mn in **2**.

After transition metals have been precisely located with full occupancies in the [Mn­(ptac)_3_] and [Co­(acac)_3_] units, X-ray fluorescence spectroscopy^51,52^ was carried out for diastereomers **1** and **2** to confirm the oxidation states of the Co and Mn ions (Figure 5). This element-specific method with high chemical sensitivity allows us to distinguish different oxidation states of the probed elements. The fluorescence spectrum globally shifts toward higher energy with the increase of the element's formal oxidation state. By comparison of the element *K-*edge in the structure under investigation with *K-*edges in the standards of the same element with a similar coordination environment, the formal oxidation state can be determined. The X-ray fluorescence spectra of both diastereomers **1** and **2**, as well as Co and Mn standards with different oxidation states, were recorded using a synchrotron radiation source. The *K-*edge values were extracted from the first-order derivative of each spectrum. It was found that both isomers display the same Co *K-*edge energy (7751 eV) as in its trivalent standard, [Co^III^(acac)_3_], as well as the same Mn *K-*edge energy (6563 eV) as in its divalent standard, [NaMn^II^(ptac)_3_], clearly confirming the presence of Co^III^ and Mn^II^ ions in both heterometallic pentanuclear diastereomers **1** and **2**, respectively (see the Supporting Information, pages S10–11 for detailed experimental procedures).

**Figure 5:**
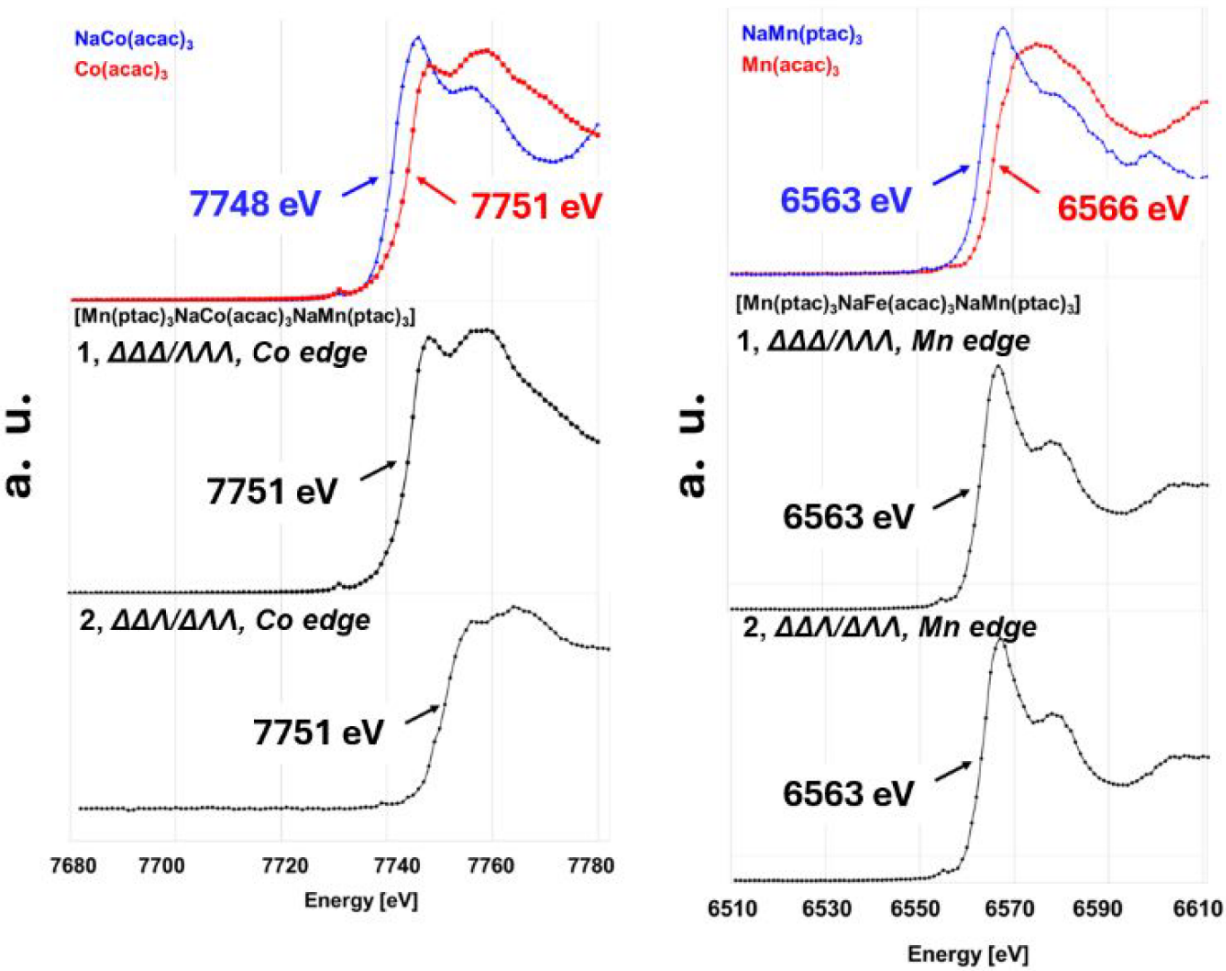
X-ray fluorescence scans collected in steps of 1 eV at 100 K around: the Co *K*-edge for the crystalline powder of [NaCo^II^(acac)_3_] (top-left, blue) and [Co^III^(acac)_3_] (top-left, red) compared with the anomalous scattering factor *f*′ plots of Co position in the structures of **1** (center-left) and **2** (bottom-left); the Mn *K*-edge for the crystalline powder of [NaMn^II^(ptac)_3_] (top-right, blue) and [Mn^III^(acac)_3_] (top-right, red) compared with the anomalous scattering factor *f*′ plots of Mn position in the structures of **1** (center-right) and **2** (bottom-right).

The above analysis of diastereomers **1** and **2** confirms the exact location of Co and Mn ions as well as their Co^III^ and Mn^II^ oxidation states to define the pentanuclear diastereomers as [Mn^II^(ptac)_3_NaCo^III^(acac)_3_NaMn^II^(ptac)_3_]. The search of the CCDC database^60^ clearly indicates that the vast majority of the heterometallic Co–Mn complexes have been defined as Co^III^/Mn^II^ pair, while a few compounds do appear as Co^II^/Mn^III^, including some with chelating ligands.^61^

### Properties of Diastereomers **1** and **2**

Diastereomers should have different physical properties (i.e., melting point, volatility, solubility),^6^ as well as chemical properties (i.e., stability, sensitivity, reactivity).^28,62^ We compared the properties of diastereomers **1** and **2** that are most closely related to their potential applications as single-source precursors for oxide cathode materials.

Diastereomers **1** and **2** share the same green color but can be easily distinguished by their crystal shapes, prisms, and plates, respectively. They were found to retain their crystallinity and appear stable in open air for weeks, as confirmed by X-ray powder diffraction. Both diastereomers show good solubility in common organic solvents such as alkanes, haloalkanes, ketones, and alcohols, while coordinating solvents such as THF or ethanol break the pentanuclear assemblies into [Co­(acac)_3_] and [NaMn­(ptac)_3_] fragments (Supporting Information, Figure S5). Diastereomer **1** displays volatility and can be sublimed under the static vacuum in a sealed ampule at as low as 90 °C, while it starts to decompose at around 110 °C, with the color changing to brown and crystallinity being lost. Diastereomer **2** does not exhibit any volatility under static vacuum, perhaps due to its decomposition temperature being ca. 85 °C under these conditions. However, both diastereomers display a good volatility under dynamic vacuum (coldfinger) at ca. 70 °C, showing no structural changes as confirmed by the powder X-ray diffraction of deposits.

Direct Analysis in Real Time (DART) mass spectrometry has been successfully utilized to confirm the composition of heterometallic ions through their isotope distribution patterns, as well as to analyze the oxidation states of constituent transition metals.^51,52,63^ However, the mass spectra cannot be used to effectively distinguish the two diastereomers. Mass spectra of **1** and **2** in positive mode (Supporting Information, Figures S6 and S7) are quite similar: the “heterometallic peaks” [M-ptac]^+^ (M = [Na_2_Mn_2_Co­(acac)_3_(ptac)_6_], meas/calcd = 1487.247/1487.239, Figure 6a,c) and [M-acac]^+^ (meas/calcd = 1583.270/1583.258, Figure 6b,d) can be clearly identified along with their characteristic isotope distribution patterns in good agreement with the simulated ones. The latter unambiguously confirms the presence of hetero*tri*metallic pentanuclear molecules in the gas phase. Importantly, all homometallic fragment peaks in the mass spectra of **1** and **2** (Supporting Information, Tables S8 and S9) correspond to the oxidation states of Co and Mn as +3 and +2, respectively.

**Figure 6:**
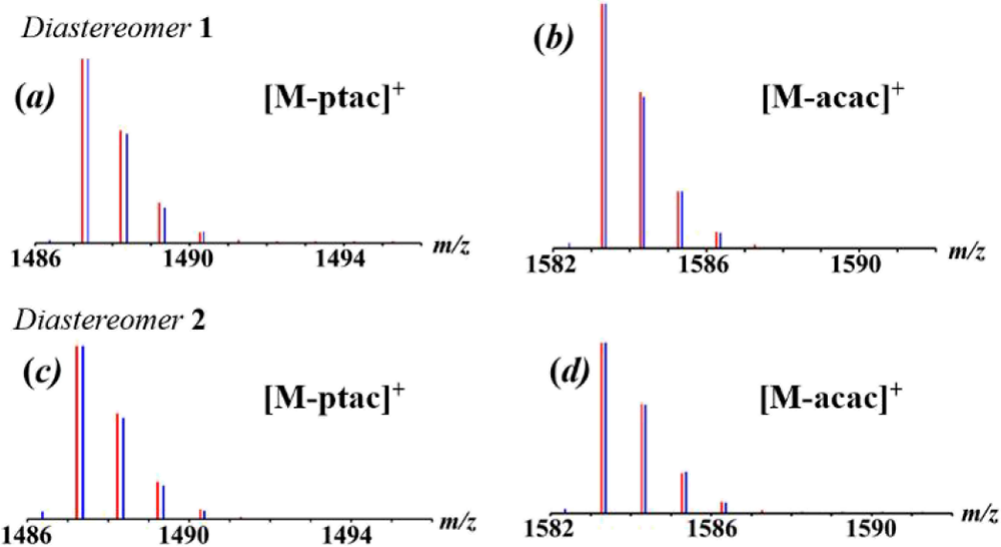
Isotope distribution patterns in the positive mode DART mass spectra of (a) [M–ptac]^+^ ion (M = [Na_2_Mn_2_Co­(acac)_3_(ptac)_6_]) in **1**; (b) [M–acac]^+^ ion in **1**; (c) [M–ptac]^+^ ion in **2**; (d) [M–acac]^+^ ion in **2**; Blue and red lines represent experimental and calculated patterns, respectively.

TGA analysis (Figure 7) confirmed the differences in the volatility and decomposition temperature of diastereomers **1** and **2**. Diastereomer **1** shows a slight mass loss due to sublimation between 90 and 110 °C before starting to decompose, while another diastereomer directly starts losing weight at around 70 °C with no apparent sublimation based on thermal analysis under the static vacuum. The weight loss curve is sharp for both diastereomers between 120 and 350 °C. The continuous weight drop at higher temperatures likely indicates the loss of Na, as it has been previously observed for a number of heterometallic compounds.^44,52^ Powder X-ray diffraction analysis revealed that the products of thermal decomposition of **1** and **2** at 450 °C in open air for 24 h are both P2–Na_0.67_Mn_0.67_Co_0.33_O_2_ cathode materials (Figure 8).^64–66^ While the diffraction intensities of the resulting quaternary oxide appear different, the crystallinities of the target phase are nearly the same for the one obtained by pyrolysis of **1** compared to that of **2**.

**Figure 7:**
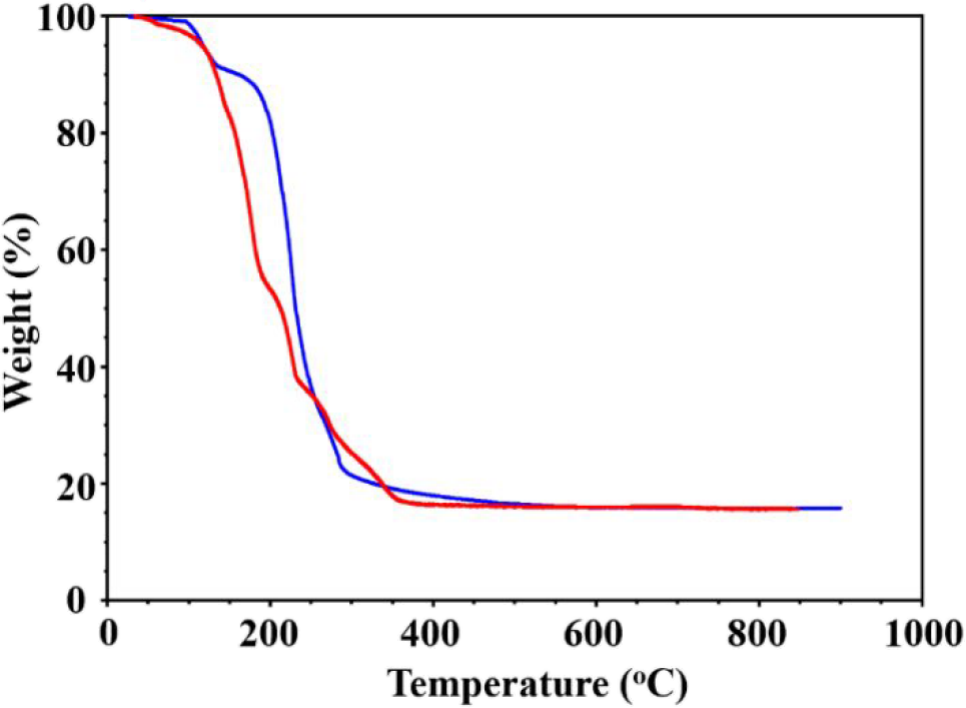
TGA plots of diastereomers **1** (blue) and **2** (red) recorded at a heating rate of 0.5 °C/min under a 25 mL/min argon protection flow.

**Figure 8:**
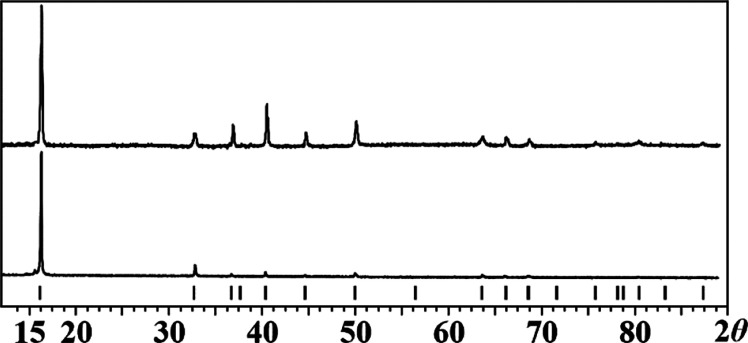
Powder X-ray diffraction patterns of the residues obtained upon decomposition of diastereomers **1** (top) and **2** (bottom) at 450 °C for 24 h in open air. Peak positions of P2–Na_0.67_Mn_0.67_Co_0.33_O_2_ from the PDF-2 database are shown as black bars at the bottom.

### Diastereomerization of **1** and **2**

The possible interconversion between two diastereomers has been studied in the solid-state, gas phase, and solution environments. First, there is no transformation between **1** and **2** detected in the solid-state (crystal-to-crystal) as monitored by the powder X-ray diffraction technique upon heating up both isomers for a prolonged time under anaerobic conditions at temperatures slightly below their respective decomposition points (100 °C for **1** and 75 °C for **2**). Second, no transformation has also been observed upon crystallization from the gas phase by checking the powder X-ray diffraction patterns of crystalline deposits obtained upon subliming diastereomer **1** under static or dynamic vacuum at different temperatures.

The transformation between two diastereomers was observed in solutions of noncoordinating solvents only, since both heterometallic structures are destroyed in coordinating solvents as described above. The transformation from **1** to **2** clearly takes place in a solution of hexanes. Dissolving **1** into dry, deoxygenated hexanes and stirring it at room temperature results in the complete transformation to diastereomer **2**. X-ray powder diffraction analysis of the solid residue obtained upon solvent evaporation unambiguously confirmed the complete transformation of **1** to **2** after 24 h. Importantly, at any time before completion, the two diastereomers were the only crystalline products detected (Figure 9). The transformation from **1** to **2** was also found to take place in alkanes, such as pentanes and cyclohexane. In contrast, after 12 h stirring in dry, deoxygenated 1,2-dichloroethane at room temperature, diastereomer **2** was shown to be completely converted to **1** as confirmed by powder X-ray diffraction of the residues isolated upon solvent evaporation (Figure 10). Similarly to the reverse transformation, only two diastereomers have been detected in the powder patterns. It should be noted that other simple haloalkanes such as dichloromethane or chloroform can also facilitate the transformation from **2** to **1**, though, according to X-ray powder diffraction analysis, the products always contain [Co­(acac)_3_], thus indicating a partial decomposition/dissociation during the process.

**Figure 9:**
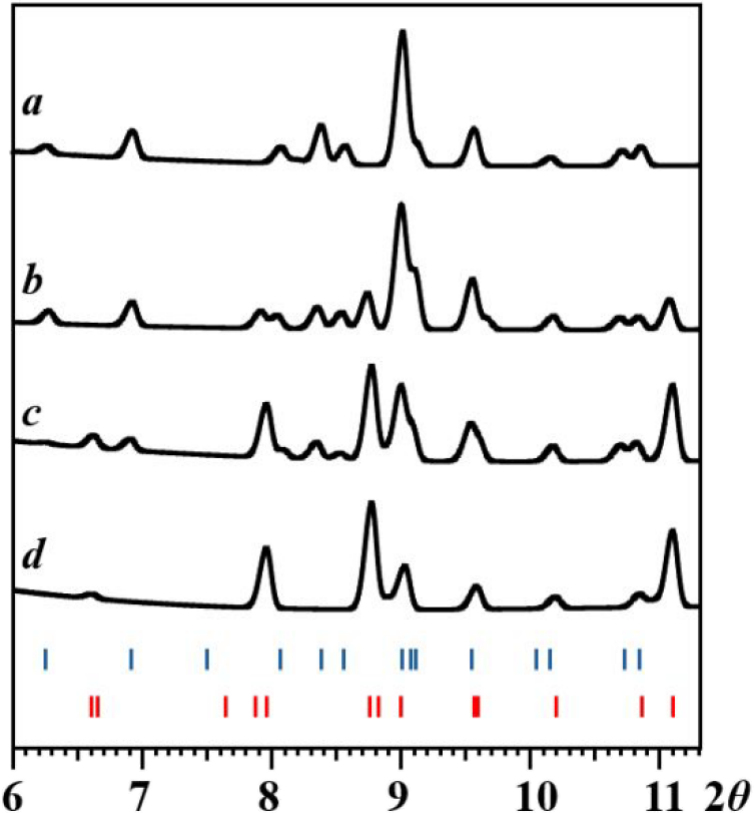
X-ray powder diffraction patterns (2θ = 6–11°) of the residues obtained after evaporation of solvent upon dissolving diastereomer **1** in hexanes at room temperature: (a) right after dissolution; (b) after 1 h; (c) after 3 h; (d) after 1 day. The theoretical peak positions of diastereomers **1** and **2** are marked with blue and red bars at the bottom, respectively.

**Figure 10:**
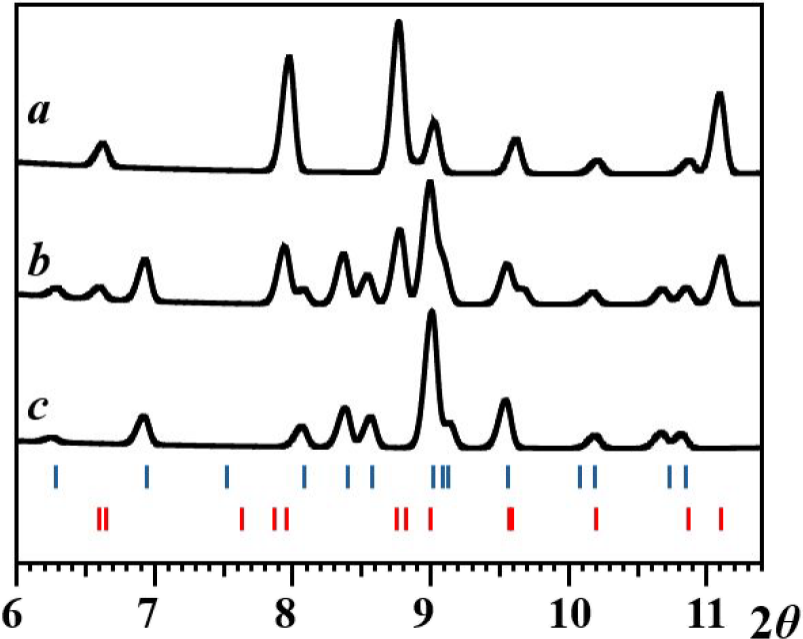
X-ray powder diffraction patterns (2θ = 6–11°) of the residues obtained after evaporation of solvent upon dissolving diastereomer **2** in 1,2-dichloroethane at room temperature: (a) right after dissolution; (b) after 1 h; (c) after 12 h. The theoretical peak positions of diastereomers **1** and **2** are marked as blue and red bars at the bottom, respectively.

Molecular simulation indicates that the diastereomerization between **1** and **2** requires at least two Mn–O­(ptac) bonds being broken and two new Mn–O­(ptac) bonds being formed (if one does not consider the dissociation/association of the [Mn­(ptac)_3_]^−^ fragments), which is far from a straightforward process. The solubility seems not to be a factor here, since both diastereomers have good solubilities in both alkanes and haloalkanes. Apparently, the significant difference between alkanes and haloalkanes is polarity, which is also an important distinction between the two diastereomers. Therefore, it seems fitting that the driving force for the transformations between **1** and **2** is the polarity of the solvent medium, with more polar diastereomer **1** being stable in haloalkanes, while less polar diastereomer **2** is preferred in alkanes.

## Conclusions

Herein, we report two out of three possible diastereomeric pairs in the pentanuclear hetero*tri*metallic assembly [Mn^II^(ptac)_3_–Na-Co^III^(acac)_3_–Na-Mn^II^(ptac)_3_]. To explore the diastereomers in heterometallic complexes, we have reasonably selected this pentanuclear assembly as a target (Scheme 2) and carefully investigated this system under different preparation conditions and crystal growth procedures. By proper control of the solvents, reaction times, and, most importantly, by monitoring the bulk products by powder X-ray diffraction, we have managed to isolate and characterize two diastereomers. Diastereomers **1** and **2** were found to crystallize in different space groups and crystal shapes while exhibiting dissimilar properties such as volatility and thermal stability. Both diastereomers can act as single-source precursors for the P2-type Na_0.67_Mn_0.67_Co_0.33_O_2_ cathode material (Figure 8).

As of today, there are numerous heterometallic compounds with chelating ligands that contain two or more chiral centers. A number of factors can be listed to rationalize the search for diastereomers in heterometallic assemblies. The difference in polarities appears to be an important one, as can be concluded from the results of this work. Another major contributor is flexibility in transition metal center coordination within polynuclear assembly. The latter can result from several compositional features that increase an asymmetry such as (i) high-spin Mn^II^ vs other divalent 3*d* transition metal ions, (ii) Mn^III^ ion with the Jahn–Teller effect; (iii) long bridges between chiral centers through linkers such as “naked” ions; (iv) the simultaneous presence of 3*d* and 4/5*d* transition metals with very different M–O bond lengths; (v) incorporation of metal ions (Pb, Bi, Ba, Ln) with large radius and unsymmetric ligand environment.

In direct connection to the present study, we can predict the existence of diastereomeric pairs in two large families of heterometallic diketonates: dinuclear [M^II^(hfac)_2_M^III^(acac)_3_][Bibr ref45] (Scheme 5, top) and trinuclear [M^II^(hfac)_2_M^III^(acac)_3_M^II^(hfac)_2_][Bibr ref38] (Scheme 5, bottom). Similar to the case depicted in Scheme 2, in the structures reported so far, the chiralities of the common element appear to be different: Λ,Δ (Δ,Λ) in dinuclear assemblies and Δ,Δ (Λ,Λ) in trinuclear assemblies (Scheme 5). Again, the title “fragment” can exist as diastereomeric and is not affected by the steric constraints that may prevent the appearance of different configurations in these molecules. These assemblies should have two and three diastereomeric pairs, respectively, while currently, there is only one configuration revealed for each (Scheme 5). Though different from the structures of **1** and **2** reported in this work by not featuring the middle “naked” Na ions between chiral centers, these molecules are known to accommodate a great variety of 3–5*d* metal ions with different M–O bond lengths.

**Scheme 5:**
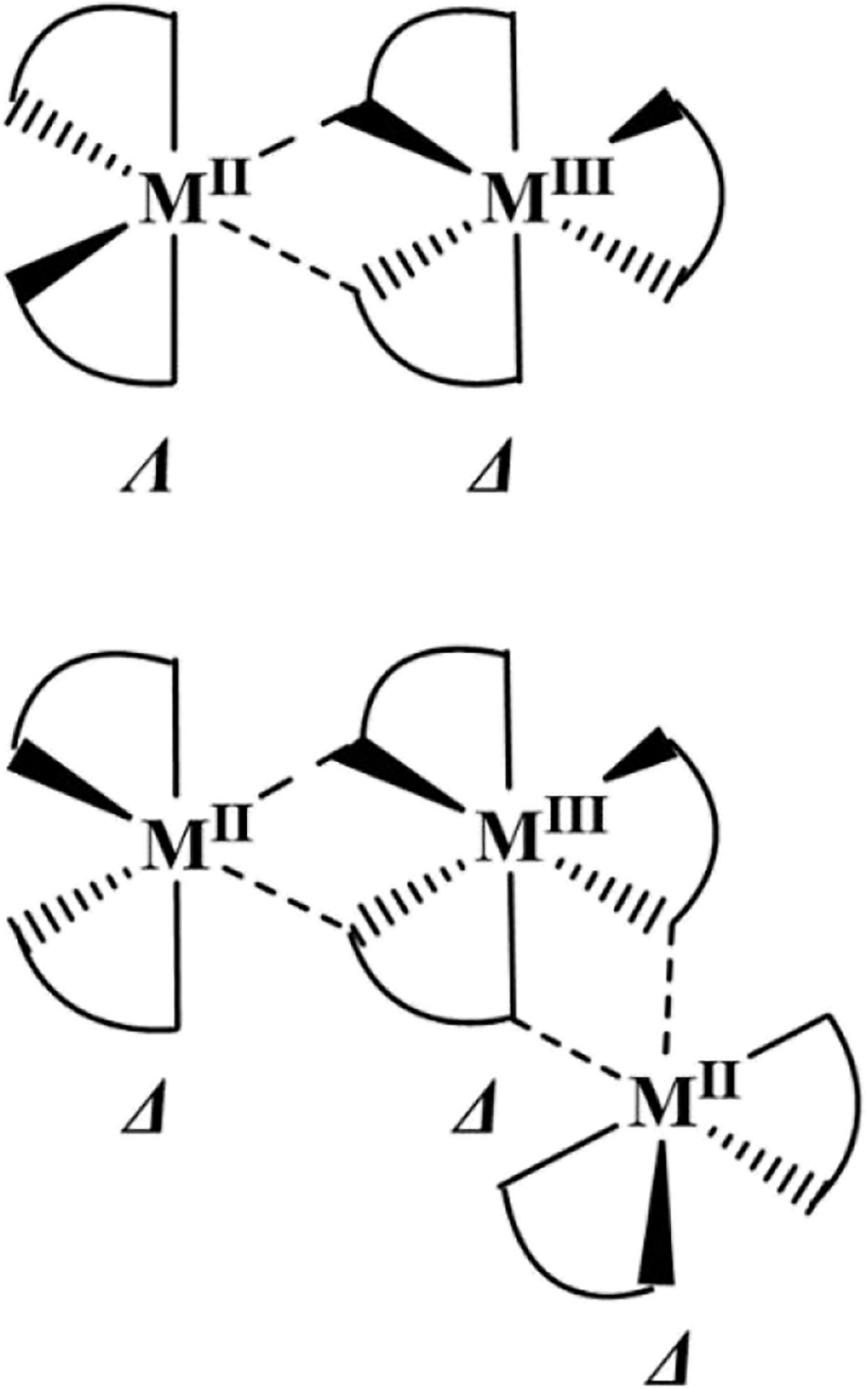
Chiralities of the Common Fragment in Dinuclear [M^II^(hfac)_2_M^III^(acac)_3_] (Top) and in Trinuclear [M^II^(hfac)_2_M^III^(acac)_3_M^II^(hfac)_2_] (Bottom) Molecules

## Experimental Section

All of the manipulations were carried out in a dry, oxygen-free argon atmosphere by employing standard Schlenk and glovebox techniques. 1,1,1-trifluoro-5,5-dimethyl-2,4-hexanedione (Hptac) was purchased from Sigma-Aldrich and used as received after checking its ^1^H NMR spectrum. Anhydrous manganese­(II) chloride (MnCl_2_), anhydrous cobalt­(II) chloride (CoCl_2_), cobalt­(III) acetylacetonate (Co­(acac)_3_), and sodium methoxide (NaOMe) were purchased from Sigma-Aldrich and used as received after checking their powder X-ray diffraction patterns. The ICP-OES analysis was carried out on an ICPE-9820 plasma atomic emission spectrometer, Shimadzu. The DART mass spectra were recorded on a JEOL AccuTof 4G LC-plus DART mass spectrometer over the mass range of *m*/*z* 50–2000 at one spectrum per second with a gas heater temperature of 200 °C. X-ray powder diffraction data were collected on a Rigaku multipurpose θ–θ X-ray SmartLab SE diffractometer (Cu Kα radiation, HyPix-400 two-dimensional advanced photon counting hybrid pixel array detector, step of 0.01° 2θ, 20 °C). Le Bail fit for powder diffraction patterns has been performed using the TOPAS version 4 software package (Bruker AXS, 2006). Thermogravimetric (TGA) measurements were carried out under 25 mL/min argon protection flow at a heating rate of 0.1–1 °C/min using a TGA 5500 (TA Instruments-Waters LLC).

Single-crystal X-ray diffraction data for complexes **1** and **2** were collected using 30 keV synchrotron radiation at the APS (Beamline 15-ID-D) with a Huber 4-circle diffractometer and a PILATUS3 detector. Data were processed with SAINT and SADABS, and structures were solved with SHELXT and refined with SHELXL via OLEX2. Non-hydrogen atoms were refined anisotropically; hydrogen atoms were placed in idealized positions. Disorder was treated with anisotropic refinement and standard SHELX restraints.

Synchrotron X-ray resonant diffraction measurements of **1** and **2** were studied by using synchrotron X-ray diffraction at two energies (near Co and Mn *K*-edges) at APS (Beamline 15-ID-D). Data were processed with SAINT and SADABS, structures solved with SHELXT and refined in OLEX2 using SHELXL. Anomalous dispersion data were used to refine Mn/Co occupancies, with fixed geometry from 30 keV data. Difference maps and site occupancies were determined using multiwavelength refinement.

Fluorescence X-ray absorption scans were collected for **1** and **2** at the Mn and Co *K*-edges by using a Vortex-60EX detector. Mn^3+^/[Mn­(acac)_3_] and Mn^2+^/[NaMn­(ptac)_3_], as well as Co^3+^/[Co­(acac)_3_] and Co^2+^/[NaCo­(acac)_3_], were used as standards. *K*-edge energies were determined from the first and second derivatives of the spectra.

## Supplementary Material



## Notes

Experimental details, synthesis, ICP-OES data, X-ray powder diffraction patterns, crystal growth conditions, X-ray crystallographic procedures, synchrotron X-ray resonant diffraction and X-ray fluorescence spectroscopy protocols, single-crystal structures with bond distances and angles, DART mass spectra, and powder X-ray diffraction patterns of thermal decomposition products (PDF)
