# Supplementary material for: Delta/Lambda Chirality: From Enantiomers to Diastereomers in Heterometallic Complexes with Chelating Ligands
Source: Inorg Chem. 2025 Jun 20;64(26):13318–28. doi: 10.1021/acs.inorgchem.5c01670 (PMC12239082; doi:10.1021/acs.inorgchem.5c01670)
Supplement: Supplementary file 1 [file ic5c01670_si_001.pdf]

Supporting Information

Delta/Lambda Chirality: From Enantiomers to Diastereomers in Heterometallic Complexes with Chelating Ligands

Yuxuan Zhang[a], Haixiang Han[b], Zheng Wei[a], Muhammad Zulqarnain[a], Tieyan Chang[c], Yu-Sheng Chen[c], and Evgeny V. Dikarev[,a]*

[a] *Department of Chemistry, University at Albany, SUNY, Albany, NY 12222, United States*

[b] *School of Material Science and Engineering, Tongji University, Shanghai, 201804, China*

[c] *NSF's ChemMatCars, Center for Advanced Radiation Source, The University of Chicago, Argonne, IL 60439, United States*

*Author to whom correspondence should be addressed. E-mail : edikarev@albany.edu. Phone : (518)442-4401. Fax: (518)442-3462.

Table of Contents

1. Experimental Section. General Procedures........ 3

2. Synthesis of Heterometallic Complexes........ 4

3. X-Ray Powder Diffraction Patterns of Diastereomers **1** and **2**........ 5

4. Crystal Growth of Diastereomers **1** and **2**........ 7

5. X-Ray Crystallographic Procedures for Diastereomers **1** and **2**........ 8

6. X-Ray Single Crystal Refinement of Different Assignments of Co and Mn Site Occupancy in Diastereomers **1** and **2**........ 12

7. Solid State Structures of Diastereomers **1** and **2**........ 13

8. Powder X-ray Diffraction Patterns of the Residues Obtained upon Dissolving Diastereomers in Coordinating Solvents........ 17

9. Direct Analysis in Real Time (DART) Mass Spectra of Diastereomers **1** and **2**........ 18

10. Synthesis, Crystallization, and Structural Analysis of [$Co^{II}(ptac)_3NaCo^{III}(acac)_3NaCo^{II}(ptac)_3$] and [$NaMn^{II}(ptac)_3$] Complexes........ 20

11. References........ 27

1. Experimental Section. General Procedures

All of the manipulations were carried out in a dry, oxygen-free argon atmosphere by employing standard Schlenk and glove box techniques. 1,1,1-trifluoro-5,5-dimethyl-2,4-hexanedione (Hptac) was purchased from Sigma-Aldrich and used as received after checking its ^{1}H NMR spectrum. Anhydrous manganese(II) chloride ($MnCl_2$), anhydrous cobalt(II) chloride, cobalt(III) acetylacetonate ($Co(acac)_3$), and sodium methoxide (NaOMe) were purchased from Sigma-Aldrich and used as received after checking their powder X-ray diffraction patterns. The ICP-OES analysis was carried out on ICPE-9820 plasma atomic emission spectrometer, Shimadzu. The DART mass spectra were recorded on a JEOL AccuTof 4G LC-plus DART mass spectrometer over the mass range of *m/z* 50–2000 at one spectrum per second with a gas heater temperature of 200 °C. X-ray powder diffraction data were collected on a Rigaku multipurpose θ-θ X-ray SmartLab SE diffractometer (Cu Kα radiation, HyPix-400 two-dimensional advanced photon counting hybrid pixel array detector, step of 0.01° 2θ, 20 °C). Le Bail fit for powder diffraction patterns has been performed using TOPAS version 4 software package (Bruker AXS, 2006). Thermogravimetric (TGA) measurements were carried out under 25 mL/min argon protection flow at a heating rate of 0.1-1 °C/min using a TGA 5500 (TA Instruments-Waters LLC).

2. Synthesis of Heterometallic Complex

Na(ptac). A flask was charged with NaOMe (0.592 g, 15.59 mmol) under dry argon atmosphere, and 30 mL of dry, oxygen-free methanol were added. Upon stirring under argon protection, Hptac (3.03 g, 3.00 mL) was slowly added to the solution. The colorless, transparent solution was stirred for one hour at room temperature followed by evaporation of the solvent under vacuum. The final white product was isolated by further drying the residue overnight under vacuum at 100 °C. Yield is 2.932 g (94%). The complex is soluble in polar solvents such as THF, alcohols and ketones. ^{1}H NMR (500 MHz, $CDCl_3$, 20 °C): δ = 1.40 (s, -C(C*H*$_3$)$_3$), 4.40 (s, -C*H*).

***ΔΔΔ/ΛΛΛ*-[Mn(ptac)$_3$NaCo(acac)$_3$NaMn(ptac)$_3$] (1)**

A flask was charged with a mixture of anhydrous Co(acac)3 (35.1 mg, 0.099 mmol) and [NaMn(ptac)3] (131 mg, 0.197 mmol) under argon atmosphere, and 20 mL of dry, oxygen-free 1,2-dichloroethane was added. The solution was stirred for 2 days at room temperature to result in the color change from yellow-greenish to green. The solvent was then evaporated under vacuum at room temperature, and the green residue was dried under vacuum at 50 oC sand bath overnight. The yield is ca. 163 mg (98%). ICP-OES (2% HNO_3 water solution, 20 °C): Na, 2.80% (Calcd: 2.73%); Mn, 6.60% (Calcd: 6.53%); Co, 3.50% (Calcd: 3.50%). Mass-spectrometry (DART-MS): [M-ptac]$^+$ (meas/calcd = 1487.247/1487.239), [M-acac]$^+$ (meas/calcd = 1583.270/1583.258), M = [Na_2Mn_2Co(acac)$_3$(ptac)$_6$].

***ΔΔΛ/ΛΛΔ*-[Mn(ptac)$_3$NaCo(acac)$_3$NaMn(ptac)$_3$] (2)**

A flask was charged with a mixture of anhydrous Co(acac)$_3$ (35.1 mg, 0.099 mmol) and [NaMn(ptac)$_3$] (131 mg, 0.197 mmol) under argon atmosphere, and 20 mL of dry, oxygen-free hexanes was added. The solution was stirred for 2 days at room temperature to result in the color change from yellow-greenish to green. The solvent was then evaporated under vacuum at room temperature, and the green residue was dried under vacuum at 50 ^{o}C sand bath overnight. The yield is *ca.* 163 mg (98%). ICP-OES (2% HNO_3 water solution, 20 °C): Na, 2.90% (Calcd: 2.73%); Mn, 6.70% (Calcd: 6.53%); Co, 3.50% (Calcd: 3.50%). Mass-spectrometry (DART-MS): [M-ptac]$^+$ (meas/calcd = 1487.247/1487.239) and [M-acac]$^+$ (meas/calcd = 1583.271/1583.258), M = [Na_2Mn_2Co(acac)$_3$(ptac)$_6$].

3. X-Ray Powder Diffraction Patterns of Diastereomers 1 and 2

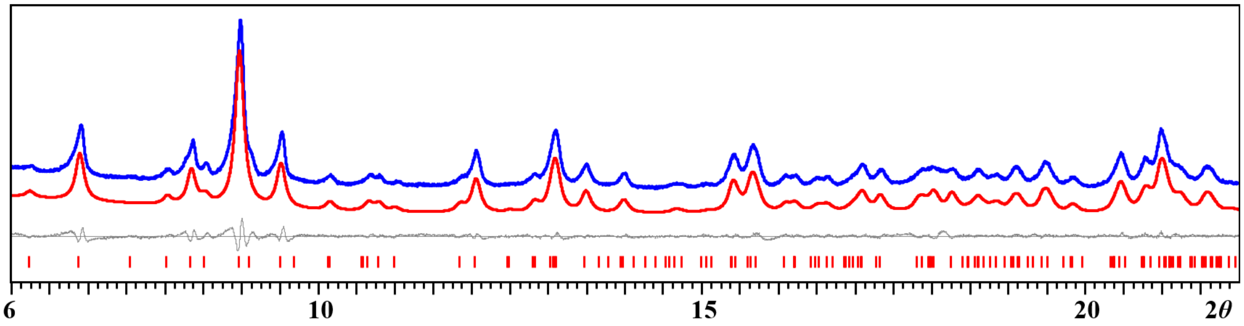

Figure S1. X-ray powder diffraction pattern and Le Bail fit for the bulk powder of diastereomer **1**. Blue and red curves are experimental and calculated patterns, respectively, grey curve is the difference line. Theoretical peak positions are marked at the bottom as red bars.

Table S1. Unit Cell Parameters of Diastereomer **1** Obtained from the Le Bail Fit and from the Single Crystal Data.

**$\Delta\Delta\Delta/\Lambda\Lambda\Lambda$-Mn(ptac)$_3$NaCo(acac)$_3$NaMn(ptac)$_3$ (1)**		
	Le Bail Fit (20 °C)	Single Crystal Data (-173 °C)
Space Group	*P*-1	*P*-1
a (Å)	13.1788(3)	12.8693(13)
b (Å)	17.0329(3)	16.8504(18)
c (Å)	19.6802(5)	19.450(2)
α (°)	74.626(2)	74.511(3)
β (°)	77.558(3)	75.420(4)
γ (°)	83.8654(17)	82.541(3)
V (Å^3)	4153.76(16)	3924.8(7)

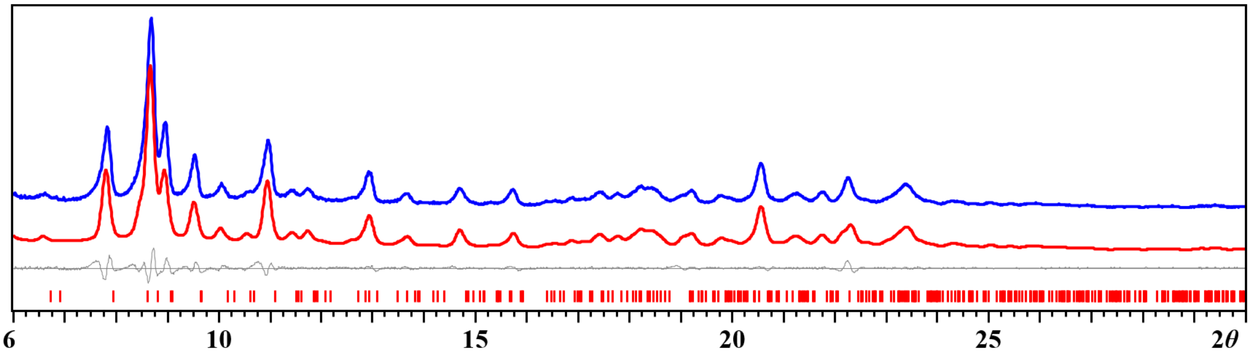

Figure S2. X-ray powder diffraction pattern and Le Bail fit for the bulk powder of diastereomer **2**. Blue and red curves are experimental and calculated patterns, respectively, grey curve is the difference line. Theoretical peak positions are marked at the bottom as red bars.

Table S2. Unit Cell Parameters of Diastereomer **2** Obtained from the Le Bail Fit and from the Single Crystal Data.

**ΔΔΛ/ΛΛΔ-Mn(ptac)$_3$NaCo(acac)$_3$NaMn(ptac)$_3$ (2)**		
	Le Bail Fit (20 °C)	Single Crystal Data (-173 °C)
Space Group	$P2_1/c$	$P2_1/c$
a (Å)	23.505(2)	23.5976(7)
b (Å)	20.0576(11)	19.8678(6)
c (Å)	18.3735(15)	18.1711(5)
α (°)	90	90
β (°)	108.921(9)	110.3740(10)
γ (°)	90	90
V (Å^3)	8194.2(12)	7986.2(4)

4. Crystal Growth of Diastereomers 1 and 2

Single crystals of diastereomers **1** and **2** suitable for X-ray structural measurements were obtained at low temperatures under argon atmosphere. Green, prism-shaped crystals of **1** were grown from its saturated dichloroethane solution in a sealed ampule under argon atmosphere at -20 ^oC for two weeks. Green, plate-shaped crystals of **2** were grown from its saturated hexanes solution in a sealed ampule under argon atmosphere at -20 ^oC for 3 days.

5. X-Ray Crystallographic Procedures for Diastereomers 1 and 2

The single crystal diffraction data for *ΔΔΔ/ΛΛΛ*-[Mn(ptac)$_3$NaCo(acac)$_3$NaMn(ptac)$_3$] (**1**) and *ΔΔΛ/ΛΛΔ*-[Mn(ptac)$_3$NaCo(acac)$_3$NaMn(ptac)$_3$] (**2**) were measured at 100(2) K on a Huber Kappa 4-circle diffraction system with a DECTRIS PILATUS3 X 2M(CdTe) pixel array detector using ϕ scans (synchrotron radiation at λ = 0.41328 Å) located at the Advanced Photon Source, Argonne National Laboratory (NSF's ChemMatCARS, Sector 15, Beamline 15-ID-D). The data were collected using ϕ scans. Data reduction and integration were performed with the Bruker software package SAINT (version 8.38A).[1] Data were corrected for absorption effects using the empirical methods as implemented in SADABS (version 2016/2).[2] The structures were solved by SHELXT (version 2018/2)[3] and refined by full-matrix least-squares procedures using the Bruker SHELXL (version 2018/3)[4] software package through the OLEX2 graphical interface.[5] All non-hydrogen atoms were refined anisotropically. Hydrogen atoms were included in idealized positions for structure factor calculations, with U_{iso}(H) = 1.2 U_{eq}(C) and U_{iso}(H) = 1.5 U_{eq}(C) for methyl groups. All disordered parts were modeled with anisotropic thermal parameters using similarity restraints. The displacement parameters for disordered parts were also restrained with the combination of RIGU/SIMU commands. All restraint commands were applied using their SHELXTL program default estimated standard deviations, except a deviation of 0.01 was applied for SIMU. Crystallographic data, details of the data collection and structure refinement for these structures are listed in Table S3.

Table S3. Crystal Data and Structure Refinement Parameters for Diastereomers **1** and **2**.

Compound	**1**	**2**
Empirical formula	$C_{63}H_{81}Co_1F_{18}Mn_2Na_2O_{18}$	$C_{63}H_{81}Co_1F_{18}Mn_2Na_2O_{18}$
CCDC Number	2374961	2374962
Formula weight	1683.06	1683.06
Temperature (K)	100(2)	100(2)
Wavelength (Å)	0.41328	0.41328
Crystal system	Triclinic	Monoclinic
Space group	*P*-1	$P2_1/c$
a (Å)	12.8693(13)	23.5976(7)
b (Å)	16.8504(18)	19.8678(6)
c (Å)	19.450(2)	18.1711(5)
α (°)	74.511(3)	90.00
β (°)	75.420(4)	110.3740(10)
γ (°)	82.541(3)	90.00
V (Å^3)	3924.8(7)	7986.2(4)
Z	2	4
ρ_{calcd} (g·cm^{-3})	1.424	1.400
μ (mm^{-1})	0.155	0.152
F(000)	1728	3456
Crystal size (mm)	0.025×0.028×0.032	0.070×0.080×0.120
θ range for data collection (°)	0.850-16.313	1.070-19.907
Reflections collected	150066	463568
Independent reflections	19957 [R_{int} = 0.1239]	37321 [R_{int} = 0.0671]
Transmission factors (min/max)	0.6516/0.7440	0.6371/0.6925
Data/restraints/params.	19957/0/961	37321/2253/1556
$R1,^a$ $wR2^b$ $(I > 2\sigma(I))$	0.0433, 0.1085	0.0424, 0.1089
$R1,^a$ $wR2^b$ (all data)	0.0594, 0.1179	0.0573, 0.1191
Quality-of-fit[c]	1.027	1.030

$R_{int} = \Sigma|F_o^2 - \langle F_o^2\rangle|/\Sigma|F_o^2|$

$^aR1 = \Sigma||F_o|-|F_c||/\Sigma|F_o|$. $^bwR2 = [\Sigma[w(F_o^2-F_c^2)^2]/\Sigma[w(F_o^2)^2]]$.

[c]Quality-of-fit = $[\Sigma[w(F_o^2-F_c^2)^2]/(N_{obs}-N_{params})]^{½}$, based on all data.

Synchrotron X-Ray Resonant Diffraction Procedures

Single crystals of heterotrimetallic compounds *ΔΔΔ/ΛΛΛ*-[Mn(ptac)$_3$NaCo(acac)$_3$NaMn(ptac)$_3$] and *ΔΛΛ/ΛΔΔ*-[Mn(ptac)$_3$NaCo(acac)$_3$NaMn(ptac)$_3$] were mounted on a glass fiber and cooled to 100 K using an Oxford Instruments Cryojet cryostat. The Huber Kappa 4-circle diffraction system, integrated with a DECTRIS PILATUS3 X 2M(CdTe) pixel array detector, was modified for synchrotron use at the NSF's ChemMatCARS beamline at the Advanced Photon Source (Argonne National Laboratory). Diffraction data were collected at three different energies. For *ΔΔΔ/ΛΛΛ*-[Mn(ptac)$_3$NaCo(acac)$_3$NaMn(ptac)$_3$]: 30 keV far away from absorption edges, 7.672 keV, which is slightly below the Co *K*-edge, and 6.498 keV, which is slightly below the Mn *K*-Edge. For *ΔΛΛ/ΛΔΔ*-[Mn(ptac)$_3$NaCo(acac)$_3$NaMn(ptac)$_3$]: 30 keV far away from absorption edges, 7.722 keV, which is slightly below the Co *K*-edge, and 6.516 keV, which is slightly below the Mn *K*-Edge. Data were collected using ϕ scans while manually attenuating the beam to minimize overages of individual pixels. Data reduction and integration were performed with the Bruker software package SAINT (version 8.38A).[1] Data were scaled and corrected for absorption effects using the multi-scan procedure as implemented in SADABS (version 2016/2).[2] The structures were solved by SHELXT[3] and refined by a full-matrix least-squares procedure using the Bruker SHELXL (version 2018/3)[4] software package through the OLEX2 graphical interface.[5] The scans at 30.0 keV, which is energetically well above the atomic absorption energies, gave a least-squares refinement for both models positional and displacement parameters. Crystallographic data and details of the data collection and structure refinement are listed in Table S3.

For further refinement of the Mn/Co site compositions, anomalous data sets collected at the lower-energy side of the absorption edges were used to minimize the solid-state effects neglected for calculations of dispersion factors.[6-9] The corresponding occupancies were refined using OLEX2.[5] The resonant scattering factors were calculated according to Cromer & Liberman algorithm.[10] The converged positional and displacement parameters of the final model from the 30 keV data were utilized and kept fixed while the Mn/Co occupancies of the transition metal sites were refined. Difference Fourier electron density maps at the Mn and Co *K*-edges were obtained by generating structure factor files without least-square refinements of the atomic models generated by the data set at 30 keV with the reflection data obtained at the respective metal absorption edges. The maps were visualized with the program OLEX2.[5] Simultaneous least-square refinement of the Mn and Co sites against several data sets collected at different wavelengths near-edge of the anomalous scatterers provided occupancies of Mn sites and the Co site.

X-Ray Fluorescence Spectroscopy Procedures

Single crystals of heterotrimetallic compounds *ΔΔΔ/ΛΛΛ*-[Mn(ptac)$_3$NaCo(acac)$_3$NaMn(ptac)$_3$] and *ΔΛΛ/ΛΔΔ*-[Mn(ptac)$_3$NaCo(acac)$_3$NaMn(ptac)$_3$] were selected and mounted. The beam energy was lowered to the Mn *K*-edge (6.539 keV), and fluorescence scans were collected in a step of 1 eV by using a Vortex-60EX X-ray fluorescence detector, which was calibrated using the Cu-foil. The energy region to collect for *ΔΔΔ/ΛΛΛ*-[Mn(ptac)$_3$NaCo(acac)$_3$NaMn(ptac)$_3$] was 6.505 to

6.605 keV and that for Δ,Δ,Λ-[Mn(ptac)$_3$NaCo(acac)$_3$NaMn(ptac)$_3$] was 6.510 to 6.610 keV. Next, the fluorescence scans of [Mn(acac)$_3$] single crystal (as a Mn^{3+} standard) and of [NaMn(ptac)$_3$] single crystal (as a Mn^{2+} standard) were collected in steps of 1 eV by using the Vortex-60EX X-ray fluorescence detector in the same energy regions. Finally, the beam energy was lowered to the Co *K*-edge (7.709 keV) and the fluorescence scans of *ΔΔΔ/ΛΛΛ*-[Mn(ptac)$_3$NaCo(acac)$_3$NaMn(ptac)$_3$], *ΔΔΛ/ΛΔΔ*-[Mn(ptac)$_3$NaCo(acac)$_3$NaMn(ptac)$_3$], [Co(acac)$_3$] single crystal (as a Co^{3+} standard) and of [NaCo(acac)$_3$] single crystal (as a Co^{2+} standard) were also collected in steps of 1 eV by using the Vortex-60EX X-ray fluorescence detector. The energy region to collect for Δ,Δ,Δ-[Mn(ptac)$_3$NaCo(acac)$_3$NaMn(ptac)$_3$] was 7.687 to 7.787 keV and that for Δ,Δ,Λ-[Mn(ptac)$_3$NaCo(acac)$_3$NaMn(ptac)$_3$] was 7.680 to 7.780 keV. The *K*-edges in different compounds were extracted from the first order derivative of the near edge spectra. The more accurate *K*-edge energy values were obtained by searching the crossing points in the second order derivative of the near edge spectra. The standard deviation for all *K*-edge energy values is 10^{-4} eV.

6. X-Ray Single Crystal Refinement of Different Assignments of Co and Mn Site Occupancy in Diastereomers 1 and 2

Table S4. Refinement Results for Different Assignments of Co and Mn Site Occupancy in **1**.

**M1–M2–M3**	**Mn1-Co1-Mn2**	**Mn1-Mn2-Co1**	**Co1-Mn1-Mn2**	**2/3Mn+1/3Co in each position**
R_1,[a]	4.33%	5.38%	5.36%	4.70%
wR_2[b] (all data)	11.80%	15.33%	15.28%	13.06%
Quality-of-fit[c]	1.027	1.339	1.334	1.138
Largest diff. peak/hole ($\bar{e}\cdot Å^{-3}$) around M1	-0.49	-0.5	-1.60	-0.76
Largest diff. peak/ hole ($\bar{e}\cdot Å^{-3}$) around M2	-0.58	1.73	1.74	1.07
Largest diff. peak/ hole ($\bar{e}\cdot Å^{-3}$) around M3	-0.39	-1.57	-0.39	-0.74

[a]$R1 = \Sigma||F_o|-|F_c||/\Sigma|F_o|$. [b]$wR2 = [\Sigma[w(F_o^2-F_c^2)^2]/\Sigma[w(F_o^2)^2]]$.
[c]Quality-of-fit = $[\Sigma[w(F_o^2-F_c^2)^2]/(N_{obs}-N_{params})]^{½}$, based on all data.

Table S5. Refinement Results for Different Assignments of Co and Mn Site Occupancy in **2**.

**M1 – M2 – M3**	**Mn1-Co1-Mn2**	**Mn1-Mn2-Co1**	**Co1-Mn1-Mn2**	**2/3Mn+1/3Co in each position**
R_1,[a]	4.11%	5.66%	5.59%	4.64%
wR_2[b] (all data)	11.40%	16.14%	15.63%	12.97%
Quality-of-fit[c]	1.027	1.466	1.415	1.172
Largest diff. peak/hole ($\bar{e}\cdot Å^{-3}$) around M1	-0.85	-0.83	-2.55	-1.37
Largest diff. peak/ hole ($\bar{e}\cdot Å^{-3}$) around M2	-0.83	2.49	2.46	1.38
Largest diff. peak/ hole ($\bar{e}\cdot Å^{-3}$) around M3	-0.46	-2.38	-0.52	-0.29

[a]$R1 = \Sigma||F_o|-|F_c||/\Sigma|F_o|$. [b]$wR2 = [\Sigma[w(F_o^2-F_c^2)^2]/\Sigma[w(F_o^2)^2]]$.
[c]Quality-of-fit = $[\Sigma[w(F_o^2-F_c^2)^2]/(N_{obs}-N_{params})]^{½}$, based on all data.

7. Solid State Structures of Diastereomer 1 and 2

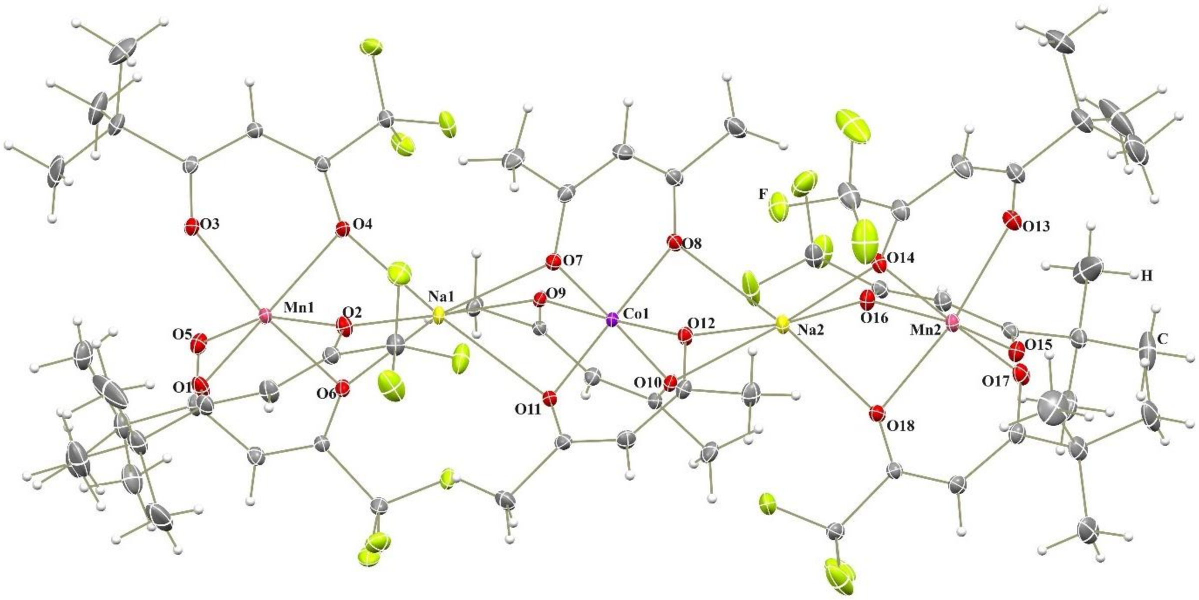

Figure S3. Solid-state structure of diastereomer **1** drawn with thermal ellipsoids at the 30% probability level. Hydrogen atoms are represented by spheres of arbitrary radius.

Table S6. Bond Distances (Å) and Angles (deg.) in the Structure of Diastereomer **1**.

**Bond distances**					
Mn(1)-O(1)	2.1168(14)	Na(1)-O(2)	2.3278(15)	Co(1)-O(7)	1.8823(14)
Mn(1)-O(2)	2.1855(14)	Na(1)-O(4)	2.4036(15)	Co(1)-O(8)	1.8862(13)
Mn(1)-O(3)	2.1039(14)	Na(1)-O(6)	2.4044(15)	Co(1)-O(9)	1.8859(13)
Mn(1)-O(4)	2.1669(13)	Na(1)-O(7)	2.4639(15)	Co(1)-O(10)	1.8847(13)
Mn(1)-O(5)	2.1380(15)	Na(1)-O(9)	2.4162(15)	Co(1)-O(11)	1.8836(13)
Mn(1)-O(6)	2.1412(14)	Na(1)-O(11)	2.5223(15)	Co(1)-O(12)	1.8821(13)
Mn(2)-O(13)	2.1323(15)	Na(2)-O(8)	2.4396(16)		
Mn(2)-O(14)	2.1894(15)	Na(2)-O(10)	2.3802(15)		
Mn(2)-O(15)	2.1203(15)	Na(2)-O(12)	2.4628(16)		
Mn(2)-O(16)	2.1541(14)	Na(2)-O(14)	2.3283(15)		
Mn(2)-O(17)	2.1023(14)	Na(2)-O(16)	2.3531(16)		
Mn(2)-O(18)	2.1874(14)	Na(2)-O(18)	2.3620(16)		

Angles					
O(1)-Mn(1)-O(2)	82.64(5)	O(2)-Na(1)-O(11)	116.89(6)	O(7)-Co(1)-O(10)	176.57(6)
O(1)-Mn(1)-O(4)	159.68(6)	O(2)-Na(1)-O(4)	72.97(5)	O(7)-Co(1)-O(11)	85.29(6)
O(1)-Mn(1)-O(5)	90.75(6)	O(2)-Na(1)-O(6)	73.65(5)	O(7)-Co(1)-O(8)	96.52(6)
O(1)-Mn(1)-O(6)	104.64(6)	O(2)-Na(1)-O(7)	113.33(5)	O(7)-Co(1)-O(9)	85.99(6)
O(3)-Mn(1)-O(1)	91.76(6)	O(2)-Na(1)-O(9)	176.82(6)	O(9)-Co(1)-O(8)	92.88(6)
O(3)-Mn(1)-O(2)	111.38(6)	O(4)-Na(1)-O(11)	170.07(6)	O(10)-Co(1)-O(8)	86.24(6)
O(3)-Mn(1)-O(4)	83.84(5)	O(4)-Na(1)-O(6)	73.89(5)	O(10)-Co(1)-O(9)	95.94(6)
O(3)-Mn(1)-O(5)	86.93(6)	O(4)-Na(1)-O(7)	116.96(5)	O(11)-Co(1)-O(10)	92.01(6)
O(3)-Mn(1)-O(6)	160.33(6)	O(4)-Na(1)-O(9)	107.65(5)	O(11)-Co(1)-O(8)	177.71(6)
O(4)-Mn(1)-O(2)	80.56(5)	O(6)-Na(1)-O(11)	106.88(5)	O(11)-Co(1)-O(9)	85.82(6)
O(5)-Mn(1)-O(2)	160.61(6)	O(6)-Na(1)-O(7)	168.05(6)	O(12)-Co(1)-O(10)	85.21(6)
O(5)-Mn(1)-O(4)	108.74(6)	O(6)-Na(1)-O(9)	109.53(5)	O(12)-Co(1)-O(11)	96.48(6)
O(5)-Mn(1)-O(6)	82.15(5)	O(7)-Na(1)-O(11)	61.53(5)	O(12)-Co(1)-O(7)	92.98(6)
O(6)-Mn(1)-O(2)	81.94(5)	O(9)-Na(1)-O(11)	62.58(5)	O(12)-Co(1)-O(8)	84.86(6)
O(6)-Mn(1)-O(4)	84.25(5)	O(9)-Na(1)-O(7)	63.54(5)	O(12)-Co(1)-O(9)	177.41(6)
O(13)-Mn(2)-O(14)	81.98(6)	O(8)-Na(2)-O(12)	62.48(5)		
O(13)-Mn(2)-O(16)	102.52(6)	O(10)-Na(2)-O(12)	63.51(5)		
O(13)-Mn(2)-O(18)	162.01(6)	O(10)-Na(2)-O(8)	64.64(5)		
O(15)-Mn(2)-O(13)	88.03(6)	O(14)-Na(2)-O(10)	167.70(6)		
O(15)-Mn(2)-O(14)	162.59(6)	O(14)-Na(2)-O(12)	104.20(5)		
O(15)-Mn(2)-O(16)	84.00(5)	O(14)-Na(2)-O(16)	77.02(5)		
O(15)-Mn(2)-O(18)	109.87(6)	O(14)-Na(2)-O(18)	74.69(5)		
O(16)-Mn(2)-O(14)	84.30(5)	O(14)-Na(2)-O(8)	110.35(6)		
O(16)-Mn(2)-O(18)	81.81(5)	O(16)-Na(2)-O(10)	115.12(6)		
O(17)-Mn(2)-O(13)	93.54(6)	O(16)-Na(2)-O(12)	173.01(6)		
O(17)-Mn(2)-O(14)	98.21(6)	O(16)-Na(2)-O(18)	74.16(5)		
O(17)-Mn(2)-O(15)	96.59(6)	O(16)-Na(2)-O(8)	110.58(6)		
O(17)-Mn(2)-O(16)	163.95(6)	O(18)-Na(2)-O(10)	109.57(5)		
O(17)-Mn(2)-O(18)	82.91(5)	O(18)-Na(2)-O(12)	112.82(6)		
O(18)-Mn(2)-O(14)	81.10(5)	O(18)-Na(2)-O(8)	173.52(6)		

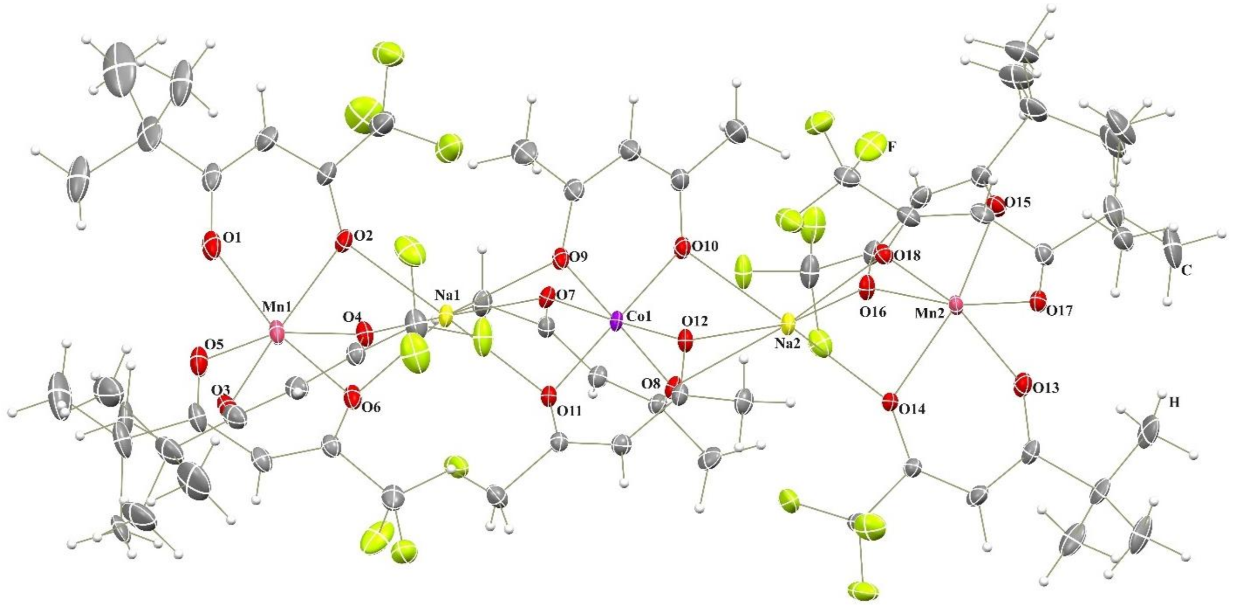

Figure S4. Solid-state structure of diastereomer **2** drawn with thermal ellipsoids at the 30% probability level. Hydrogen atoms are represented by spheres of arbitrary radius.

Table S7. Bond Distances (Å) and Angles (deg.) in the Structure of Diastereomer **2**.

Bond distances					
Mn(1)-O(1)	2.1029(11)	Na(1)-O(2)	2.3358(10)	Co(1)-O(7)	1.8821(8)
Mn(1)-O(2)	2.1825(9)	Na(1)-O(4)	2.3227(9)	Co(1)-O(8)	1.8813(8)
Mn(1)-O(3)	2.125(7)	Na(1)-O(6)	2.4104(10)	Co(1)-O(9)	1.8796(8)
Mn(1)-O(4)	2.1750(9)	Na(1)-O(7)	2.4071(9)	Co(1)-O(10)	1.8786(8)
Mn(1)-O(5)	2.1063(10)	Na(1)-O(9)	2.5085(9)	Co(1)-O(11)	1.8841(8)
Mn(1)-O(6)	2.1695(9)	Na(1)-O(11)	2.3813(9)	Co(1)-O(12)	1.8840(8)
Mn(2)-O(13)	2.1115(9)	Na(2)-O(8)	2.5267(10)		
Mn(2)-O(14)	2.1758(8)	Na(2)-O(10)	2.3932(9)		
Mn(2)-O(15)	2.115(8)	Na(2)-O(12)	2.4157(9)		
Mn(2)-O(16)	2.1510(8)	Na(2)-O(14)	2.3198(9)		
Mn(2)-O(17)	2.098(5)	Na(2)-O(16)	2.3567(9)		
Mn(2)-O(18)	2.1704(8)	Na(2)-O(18)	2.4049(9)		

Angles					
O(1)-Mn(1)-O(2)	82.83(4)	O(2)-Na(1)-O(6)	74.03(4)	O(7)-Co(1)-O(11)	85.25(4)
O(1)-Mn(1)-O(3)	98.3(3)	O(2)-Na(1)-O(7)	112.82(4)	O(7)-Co(1)-O(12)	177.48(4)
O(1)-Mn(1)-O(4)	99.82(4)	O(2)-Na(1)-O(9)	119.44(4)	O(8)-Co(1)-O(7)	96.66(3)
O(1)-Mn(1)-O(5)	93.83(4)	O(2)-Na(1)-O(11)	175.19(4)	O(8)-Co(1)-O(11)	91.99(4)
O(1)-Mn(1)-O(6)	163.21(5)	O(4)-Na(1)-O(2)	75.08(3)	O(8)-Co(1)-O(12)	85.32(4)
O(3)-Mn(1)-O(2)	163.5(3)	O(4)-Na(1)-O(6)	76.77(3)	O(9)-Co(1)-O(7)	86.21(4)
O(3)-Mn(1)-O(4)	82.2(3)	O(4)-Na(1)-O(7)	169.26(4)	O(9)-Co(1)-O(8)	176.42(4)
O(3)-Mn(1)-O(6)	98.3(3)	O(4)-Na(1)-O(9)	107.05(3)	O(9)-Co(1)-O(11)	86.11(4)
O(4)-Mn(1)-O(2)	81.29(3)	O(4)-Na(1)-O(11)	108.26(4)	O(9)-Co(1)-O(12)	91.87(4)
O(5)-Mn(1)-O(2)	108.25(4)	O(6)-Na(1)-O(9)	166.45(4)	O(10)-Co(1)-O(7)	91.92(4)
O(5)-Mn(1)-O(3)	88.2(3)	O(7)-Na(1)-O(6)	111.92(4)	O(10)-Co(1)-O(8)	85.72(4)
O(5)-Mn(1)-O(4)	164.31(4)	O(7)-Na(1)-O(9)	63.02(3)	O(10)-Co(1)-O(9)	96.35(4)
O(5)-Mn(1)-O(6)	83.93(4)	O(11)-Na(1)-O6()	103.12(4)	O(10)-Co(1)-O(11)	176.13(4)
O(6)-Mn(1)-O(2)	82.10(4)	O(11)-Na(1)-O(7)	64.37(3)	O(10)-Co(1)-O(12)	86.66(4)
O(6)-Mn(1)-O(4)	85.16(3)	O(11)-Na(1)-O(9)	63.34(3)	O(12)-Co(1)-O(11)	96.26(3)
O(13)-Mn(2)-O(14)	82.81(3)	O(10)-Na(2)-O(8)	62.57(3)		
O(13)-Mn(2)-O(15)	100.8(4)	O(10)-Na(2)-O(12)	64.94(3)		
O(13)-Mn(2)-O(16)	103.56(4)	O(10)-Na(2)-O(18)	111.01(4)		
O(13)-Mn(2)-O(18)	160.54(3)	O(12)-Na(2)-O(8)	62.12(3)		
O(15)-Mn(2)-O(14)	164.0(3)	O(14)-Na(2)-O(8)	113.50(3)		
O(15)-Mn(2)-O(16)	82.4(3)	O(14)-Na(2)-O(10)	172.28(4)		
O(15)-Mn(2)-O(18)	97.4(4)	O(14)-Na(2)-O(12)	120.00(4)		
O(16)-Mn(2)-O(14)	81.64(3)	O(14)-Na(2)-O(16)	74.43(3)		
O(16)-Mn(2)-O(18)	85.37(3)	O(14)-Na(2)-O(18)	73.72(3)		
O(17)-Mn(2)-O(13)	86.70(19)	O(16)-Na(2)-O(8)	111.48(3)		
O(17)-Mn(2)-O(14)	104.70(18)	O(16)-Na(2)-O(10)	100.45(3)		
O(17)-Mn(2)-O(15)	91.1(3)	O(16)-Na(2)-O(12)	165.36(4)		
O(17)-Mn(2)-O(16)	168.7(2)	O(16)-Na(2)-O(18)	75.94(3)		
O(17)-Mn(2)-O(18)	86.33(19)	O(18)-Na(2)-O(8)	170.48(3)		
O(18)-Mn(2)-O(14)	81.43(3)	O(18)-Na(2)-O(12)	109.22(3)		

8. Powder X-ray Diffraction Patterns of the Residues Obtained upon Dissolving Diastereomers in Coordinating Solvent

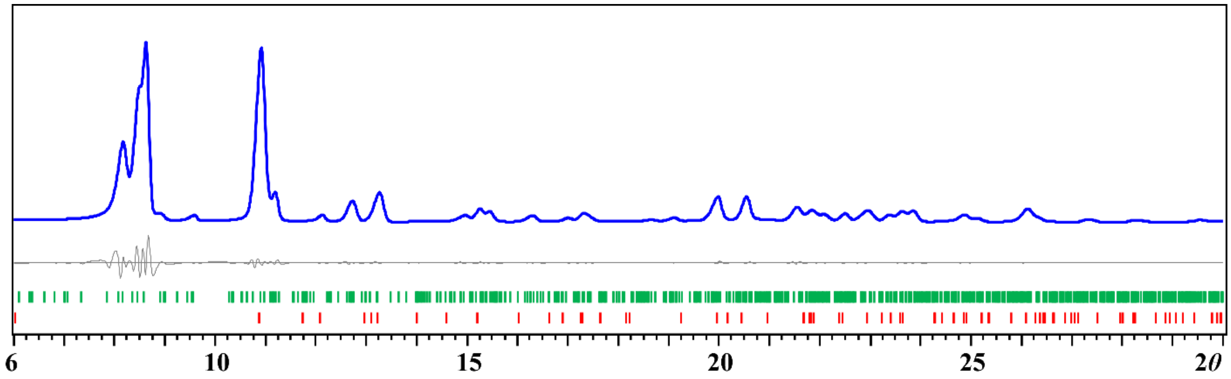

Figure S5. Powder X-ray diffraction pattern for the bulk powder of the reaction residue obtained by evaporating the solvent from acetone solution of mixture of diastereomers **1** and **2**. Blue curve is the experimental powder pattern. Calculated single peak patterns for [Co(acac)$_3$] and [NaMn(ptac)$_3$] with theoretical peak positions are shown at the bottom as red and green bars, respectively.

9. Direct Analysis in Real Time (DART) Mass Spectra of Diastereomers 1 and 2

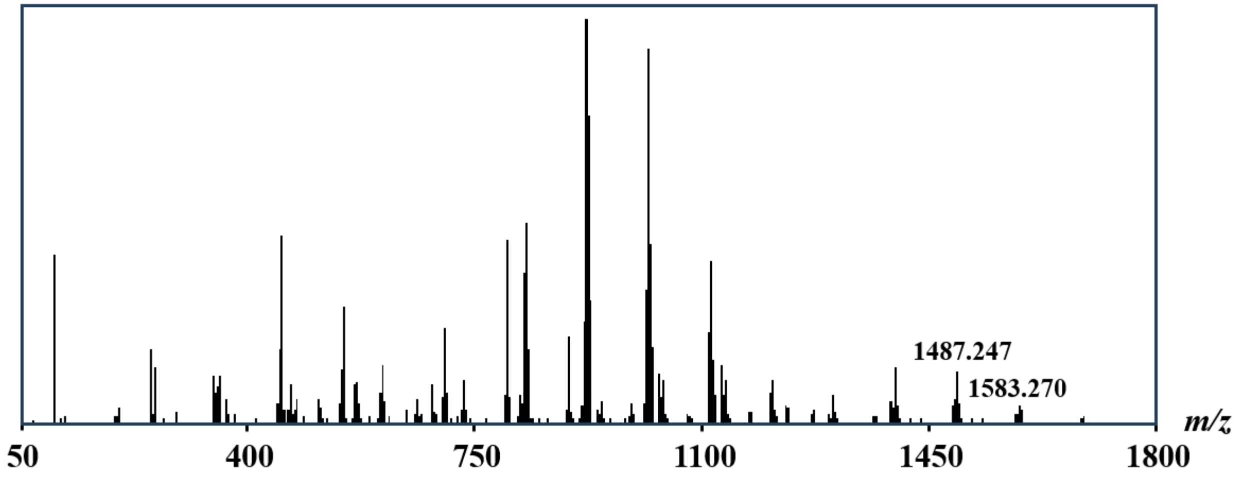

Figure S6. Positive-mode DART mass spectrum of **1**.

Table S8. Assignment of Ions Detected in Positive-Ion DART Mass Spectrum of **1** (acac = $C_5H_7O_2$, ptac = $C_8H_{10}O_2F_3$).

Ions	*Observed*	*Calculated*	*Δ*	*% Base*
**$[Na_2Mn^{II}_2Co^{III}(acac)_2(ptac)_6]^+$**	1583.2699	1583.2578	0.0121	4.6%
**$[Na_2Mn^{II}_2Co^{III}(acac)_3(ptac)_5]^+$**	1487.2470	1487.2391	0.0079	12.9%
**$[Na_2Mn^{II}_2Co^{III}(acac)_4(ptac)_4]^+$**	1391.2275	1391.2204	0.0071	14.1%
**$[Na_2Mn^{II}Co^{III}(acac)_2(ptac)_4]^+$**	1138.1989	1138.1932	0.0057	10.8%
**$[HNaMn^{II}Co^{III}(acac)_2(ptac)_4]^+$**	1115.2087	1115.2034	0.0053	40.1%
**$[Na_2Mn^{II}Co^{III}(acac)_3(ptac)_3]^+$**	1042.1787	1042.1744	0.0043	10.8%
**$[HNaMn^{II}Co^{III}(acac)_3(ptac)_3]^+$**	1019.1894	1019.1847	0.0047	92.5%
**$[HNaMn^{II}Co^{III}(acac)_4(ptac)_2]^+$**	923.1701	923.1660	0.0041	100%
**$[HNaMn^{II}Co^{III}(acac)_5(ptac)]^+$**	827.1511	827.1473	0.0038	37.1%
**$[NaCo^{III}_2(acac)_6]^+$**	735.1278	735.1238	0.0050	11.1%
**$[Na_2Mn^{II}(ptac)_3]^+$**	686.1117	686.1075	0.0042	10.0%
**$[NaMn^{II}(ptac)_2]^+$**	468.0576	468.0543	0.0033	9.8%
**$[HCo^{III}(acac)_3]^+$**	357.0769	357.0748	0.0021	11.7%
**$[Co^{III}(acac)_2]^+$**	258.0316	258.0302	0.0014	14.3%
**$[HMn^{II}(acac)_2]^+$**	254.0438	254.0429	0.0009	18.5%

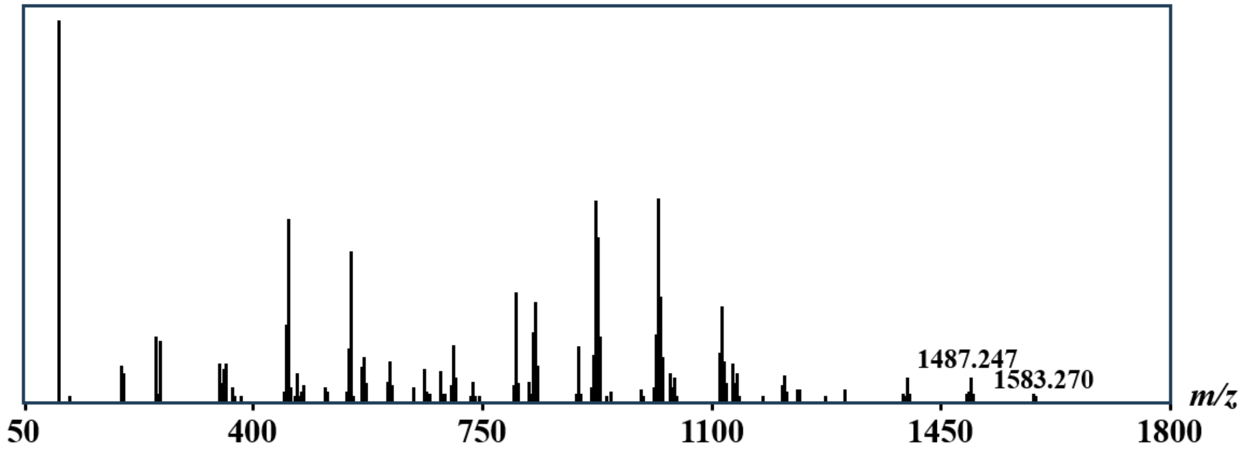

Figure S7. Positive-mode DART mass spectrum of **2**.

Table S9. Assignment of Ions Detected in Positive-Ion DART Mass Spectrum of **2** (acac = $C_5H_7O_2$, ptac = $C_8H_{10}O_2F_3$).

Ions	*Observed*	*Calculated*	*Δ*	*% Base*
**$[Na_2Mn^{II}_2Co^{III}(acac)_2(ptac)_6]^+$**	1583.2701	1583.2578	0.0123	2.5%
**$[Na_2Mn^{II}_2Co^{III}(acac)_3(ptac)_5]^+$**	1487.2467	1487.2391	0.0076	6.6%
**$[Na_2Mn^{II}_2Co^{III}(acac)_4(ptac)_4]^+$**	1391.2268	1391.2204	0.0062	6.9%
**$[Na_2Mn^{II}Co^{III}(acac)_2(ptac)_4]^+$**	1138.1979	1138.1932	0.0047	7.9%
**$[HNaMn^{II}Co^{III}(acac)_2(ptac)_4]^+$**	1115.2089	1115.2034	0.0055	25.1%
**$[Na_2Mn^{II}Co^{III}(acac)_3(ptac)_3]^+$**	1042.1791	1042.1744	0.0047	6.7%
**$[HNaMn^{II}Co^{III}(acac)_3(ptac)_3]^+$**	1019.1895	1019.1847	0.0048	53.0%
**$[HNaMn^{II}Co^{III}(acac)_4(ptac)_2]^+$**	923.1702	923.1660	0.0042	52.8%
**$[HNaMn^{II}Co^{III}(acac)_5(ptac)]^+$**	827.1511	827.1473	0.0038	18.5%
**$[NaCo^{III}_2(acac)_6]^+$**	735.1274	735.1238	0.0036	5.7%
**$[Na_2Mn^{II}(ptac)_3]^+$**	686.1115	686.1075	0.0040	8.8%
**$[NaMn^{II}(ptac)_2]^+$**	468.0580	468.0543	0.0037	7.9%
**$[HCo^{III}(acac)_3]^+$**	357.0769	357.0748	0.0021	10.2%
**$[Co^{III}(acac)_2]^+$**	258.0315	258.0302	0.0013	17.1%

10. Synthesis, Crystallization, and Structural Analysis of $[Co^{II}(ptac)_3NaCo^{III}(acac)_3NaCo^{II}(ptac)_3]$ and $[NaMn^{II}(ptac)_3]$ Compounds

[NaMn(ptac)$_3$]. A flask was charged with grinded Na(ptac) (1.040 g, 4.767 mmol) and anhydrous $MnCl_2$ (0.200 g, 1.589 mmol) powder, and 20 mL of oxygen-free acetone was added under dry argon atmosphere. The yellow-colored solution was stirred for 4 hours at room temperature to result in appearance of a white precipitate. The precipitate (NaCl) was filtered off, and the clear yellow solution was evaporated under vacuum. The yellow residue was further dried under vacuum at 100 ^{o}C sand bath overnight. The yield was *ca*. 1.001 g (95%). ICP-OES (2% HNO_3 water solution, 20 ^{o}C): Na, 3.60% (Calcd: 3.47%); Mn, 8.30% (Calcd: 8.28%).

***ΔΔΛ/ΛΛΔ*-[Co(ptac)$_3$NaCo(acac)$_3$NaCo(ptac)$_3$].** A flask was charged with a mixture of anhydrous Co(acac)$_3$ (41.2 mg, 0.116 mmol), Na(ptac) (151 mg, 0.692 mmol) and anhydrous $CoCl_2$ (30.0 mg, 0.231 mmol), and 20 mL of dry, oxygen-free hexanes was added under argon atmosphere. The solution was stirred for 2 days at room temperature resulting in the appearance of green solution and white precipitate. The solution was then filtered off and sealed in a L-shape ampule under argon atmosphere for single crystal growth. After one week, green, block-shaped single crystals suitable for single crystal X-ray measurement were crystalized at the inner side of the ampule.

X-ray Crystallographic Procedures

ΔΔΛ/ΛΛΔ-[Co(ptac)$_3$NaCo(acac)$_3$NaCo(ptac)$_3$]

Single crystal diffraction data for *ΔΔΛ/ΛΛΔ*-[Co(ptac)$_3$NaCo(acac)$_3$NaCo(ptac)$_3$] were measured at 100(2) K on a Bruker D8 VENTURE X-ray diffractometer with PHOTON 100 CMOS detector equipped with a Mo-target fine-focus sealed X-ray tube (λ = 0.71073 Å). Data reduction and integration were performed with the Bruker software package SAINT (version 8.38A).[1] Data were corrected for absorption effects using the empirical methods as implemented in SADABS (version 2016/2).[2] The structures was solved by SHELXT (version 2018/2)[3] and refined by full-matrix least-squares procedures using the Bruker SHELXL (version 2019/2)[4] software package through the OLEX2 graphical interface.[5] All non-hydrogen atoms were refined anisotropically. Hydrogen atoms were included in idealized positions for structure factor calculations, with U_{iso}(H) = 1.2 U_{eq}(C) and U_{iso}(H) = 1.5 U_{eq}(C) for methyl groups. All disordered parts were modeled with anisotropic thermal parameters using similarity restraints. The displacement parameters for disordered parts were also restrained with the combination of RIGU/SIMU commands. All restraint commands were applied using their SHELXTL program default estimated standard deviations, except a deviation of 0.01 was applied for SIMU. Crystallographic data, details of the data collection and structure refinement for the structure are listed in Table S11.

[NaMn(ptac)$_3$]

The single crystal diffraction data for [NaMn(ptac)$_3$] were measured at 100(2) K on a Rigaku XtaLAB Synergy-S X-ray diffractometer equipped with a HyPix-6000HE hybrid photon counting (HPC) detector and a microfocus Cu-Kα radiation (λ = 1.54178 Å). Data collection strategy to ensure completeness and desired redundancy were determined using CrysAlisPro.[11] Data processing was performed using CrysAlisPro program. Empirical absorption correction was applied using the SCALE3 ABSPACK scaling algorithm.[12] The structure was solved by SHELXT (version 2018/2)[3] and refined by full-matrix least-squares procedures using the Bruker SHELXTL (version 2019/2)[4] software package through the OLEX2 graphical interface.[5] All non-hydrogen atoms, including those in disordered parts, were refined anisotropically. Hydrogen atoms were included in idealized positions for structure factor calculations with U_{iso}(H) = 1.2 U_{eq}(C) and U_{iso}(H) = 1.5 U_{eq}(C) for methyl groups. All disordered parts were modeled with anisotropic thermal parameters using similarity restraints. The displacement parameters for disordered parts were also restrained with the combination of RIGU/SIMU commands. All restraint commands were applied using their SHELXTL program default estimated standard deviations, except a deviation of 0.01 was applied for SIMU. Crystallographic data, details of the data collection and structure refinement for the structure are listed in Table S11.

Table S10. Crystal Data and Structure Refinement Parameters for *ΔΔΛ/ΛΛΔ*-[Co(ptac)$_3$NaCo(acac)$_3$NaCo(ptac)$_3$] and [NaMn(ptac)$_3$].

Compound	***ΔΔΛ/ΛΛΔ*-[Co(ptac)$_3$NaCo(acac)$_3$NaCo(ptac)$_3$]**	**[NaMn(ptac)$_3$]**
Empirical formula	$C_{63}H_{81}Co_3F_{18}Na_2O_{18}$	$C_{72}H_{90}F_{27}Mn_3Na_3O_{18}$
CCDC Number	2374963	2374964
Formula weight	1691.04	1990.22
Temperature (K)	100(2)	100(2)
Wavelength (Å)	0.71073	1.54184
Crystal system	Monoclinic	Triclinic
Space group	$P2_1/c$	*P*-1
a (Å)	23.407(2)	17.4650(5)
b (Å)	19.8031(18)	24.3080(7)
c (Å)	18.0164(16)	24.6514(6)
α (°)	90.00	62.970(3)
β (°)	109.172(2)	89.328(2)
γ (°)	90.00	81.071(2)
V (Å^3)	7888.1(12)	9188.8(5)
Z	4	4
ρ_{calcd} (g·cm^{-3})	1.424	1.439
μ (mm^{-1})	0.738	4.469

$F(000)$	3472	4068
Crystal size (mm)	0.09×0.120×0.150	0.030×0.040×0.110
θ range for data collection (°)	2.699-30.588	2.567-66.998
Reflections collected	180904	78519
Independent reflections	24153 [R_{int} = 0.0366]	31766 [R_{int} = 0.1192]
Transmission factors (min/max)	0.5969/0.7238	0.7863/1.0000
Data/restraints/params.	24153/503/1156	31766/2062/2930
$R1$,[a] $wR2$[b] ($I > 2\sigma(I)$)	0.0455, 0.0917	0.1128, 0.3202
$R1$,[a] $wR2$[b] (all data)	0.0665, 0.1031	0.1627, 0.3602
Quality-of-fit[c]	1.054	1.149

$R_{int} = \Sigma|F_o^2 - \langle F_o^2\rangle|/\Sigma|F_o^2|$

[a] $R1 = \Sigma||F_o|-|F_c||/\Sigma|F_o|$. [b] $wR2 = [\Sigma[w(F_o^2-F_c^2)^2]/\Sigma[w(F_o^2)^2]]$.

[c] Quality-of-fit = $[\Sigma[w(F_o^2-F_c^2)^2]/(N_{obs}-N_{params})]^{½}$, based on all data.

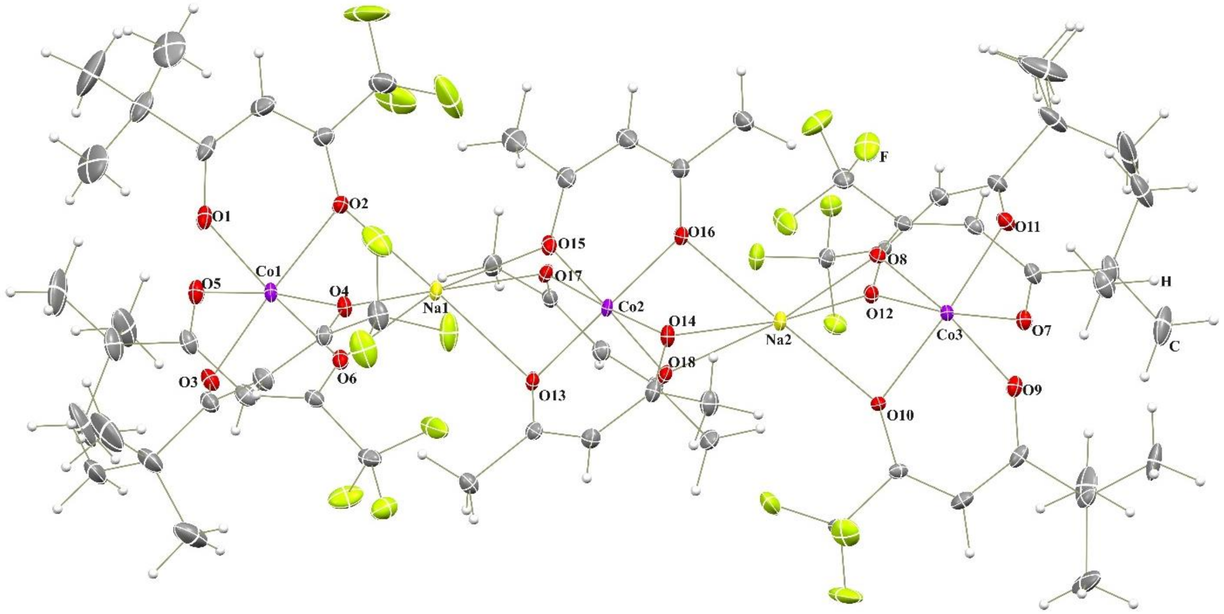

Figure S8. Solid-state structure of *ΔΔΛ/ΛΛΔ*-[Co(ptac)$_3$NaCo(acac)$_3$NaCo(ptac)$_3$] drawn with thermal ellipsoids at the 30% probability level. Hydrogen atoms are represented by spheres of arbitrary radius.

Table S11. Bond Distances (Å) and Angles (deg.) in the Structure of *ΔΔΛ/ΛΛΔ*-[Co(ptac)$_3$NaCo(acac)$_3$ NaCo(ptac)$_3$].

**Bond distances**					
Co(1)-O(1)	2.0395(14)	Co(2)-O(13)	1.8863(12)	Co(3)-O(7)	2.0496(13)
Co(1)-O(2)	2.0671(13)	Co(2)-O(14)	1.8816(13)	Co(3)-O(8)	2.0691(13)
Co(1)-O(3)	2.0596(14)	Co(2)-O(15)	1.8871(13)	Co(3)-O(9)	2.0470(13)
Co(1)-O(4)	2.0580(13)	Co(2)-O(16)	1.8845(13)	Co(3)-O(10)	2.0823(12)
Co(1)-O(5)	2.0341(13)	Co(2)-O(17)	1.8819(12)	Co(3)-O(11)	2.0489(13)
Co(1)-O(6)	2.0763(13)	Co(2)-O(18)	1.8818(12)	Co(3)-O(12)	2.0552(12)
Na(1)-O(2)	2.3384(14)	Na(2)-O(8)	2.4035(14)		
Na(1)-O(4)	2.3098(14)	Na(2)-O(10)	2.3138(14)		
Na(1)-O(6)	2.3755(15)	Na(2)-O(12)	2.3622(14)		
Na(1)-O(13)	2.3898(14)	Na(2)-O(14)	2.4074(14)		
Na(1)-O(15)	2.4861(15)	Na(2)-O(16)	2.3959(14)		
Na(1)-O(17)	2.3923(14)	Na(2)-O(18)	2.5390(14)		
**Angles**					
O(1)-Co(1)-O(2)	88.81(6)	O(13)-Co(2)-O(15)	86.47(6)	O(7)-Co(3)-O(8)	88.52(5)
O(1)-Co(1)-O(3)	90.15(6)	O(14)-Co(2)-O(13)	96.42(6)	O(7)-Co(3)-O(10)	100.60(5)
O(1)-Co(1)-O(4)	94.83(6)	O(14)-Co(2)-O(15)	91.03(6)	O(7)-Co(3)-O(12)	174.05(5)
O(1)-Co(1)-O(6)	173.70(6)	O(14)-Co(2)-O(16)	86.89(6)	O(8)-Co(3)-O(10)	84.07(5)
O(2)-Co(1)-O(6)	86.10(5)	O(14)-Co(2)-O(17)	176.55(6)	O(9)-Co(3)-O(7)	85.78(5)
O(3)-Co(1)-O(2)	171.45(6)	O(14)-Co(2)-O(18)	85.98(6)	O(9)-Co(3)-O(8)	168.85(5)
O(3)-Co(1)-O(6)	95.43(6)	O(16)-Co(2)-O(13)	175.49(6)	O(9)-Co(3)-O(10)	87.56(5)
O(4)-Co(1)-O(2)	84.64(5)	O(16)-Co(2)-O(15)	96.56(6)	O(9)-Co(3)-O(11)	93.20(6)
O(4)-Co(1)-O(3)	86.99(5)	O(17)-Co(2)-O(13)	85.52(5)	O(9)-Co(3)-O(12)	97.46(5)
O(4)-Co(1)-O(6)	88.40(5)	O(17)-Co(2)-O(15)	86.23(6)	O(11)-Co(3)-O(7)	86.32(5)
O(5)-Co(1)-O(1)	89.01(6)	O(17)-Co(2)-O(16)	91.33(6)	O(11)-Co(3)-O(8)	96.00(5)
O(5)-Co(1)-O(2)	101.10(6)	O(18)-Co(2)-O(13)	91.60(5)	O(11)-Co(3)-O(10)	173.07(5)
O(5)-Co(1)-O(3)	87.36(6)	O(18)-Co(2)-O(15)	176.25(6)	O(11)-Co(3)-O(12)	88.50(5)
O(5)-Co(1)-O(4)	173.17(6)	O(18)-Co(2)-O(16)	85.56(5)	O(12)-Co(3)-O(8)	89.09(5)
O(5)-Co(1)-O(6)	88.32(5)	O(18)-Co(2)-O(17)	96.83(5)	O(12)-Co(3)-O(10)	84.57(5)
O(2)-Na(1)-O(6)	73.74(5)	O(8)-Na(2)-O(14)	113.19(5)		
O(2)-Na(1)-O(13)	176.09(6)	O(8)-Na(2)-O(18)	171.62(5)		

O(2)-Na(1)-O(15)	118.78(6)	O(10)-Na(2)-O(8)	72.18(5)	
O(2)-Na(1)-O(17)	113.55(5)	O(10)-Na(2)-O(12)	73.07(5)	
O(4)-Na(1)-O(2)	73.38(5)	O(10)-Na(2)-O(14)	118.80(5)	
O(4)-Na(1)-O(6)	75.91(5)	O(10)-Na(2)-O(16)	174.94(6)	
O(4)-Na(1)-O(13)	108.90(5)	O(10)-Na(2)-O(18)	116.10(5)	
O(4)-Na(1)-O(15)	106.08(5)	O(12)-Na(2)-O(8)	74.75(5)	
O(4)-Na(1)-O(17)	169.35(6)	O(12)-Na(2)-O(14)	166.85(5)	
O(6)-Na(1)-O(13)	103.55(5)	O(12)-Na(2)-O(16)	102.59(5)	
O(6)-Na(1)-O(15)	167.47(6)	O(12)-Na(2)-O(18)	108.23(5)	
O(6)-Na(1)-O(17)	113.33(5)	O(14)-Na(2)-O(18)	62.44(4)	
O(13)-Na(1)-O(15)	63.99(5)	O(16)-Na(2)-O(8)	109.51(5)	
O(13)-Na(1)-O(17)	64.68(4)	O(16)-Na(2)-O(14)	65.25(5)	
O(17)-Na(1)-O(15)	63.72(5)	O(16)-Na(2)-O(18)	62.36(5)	

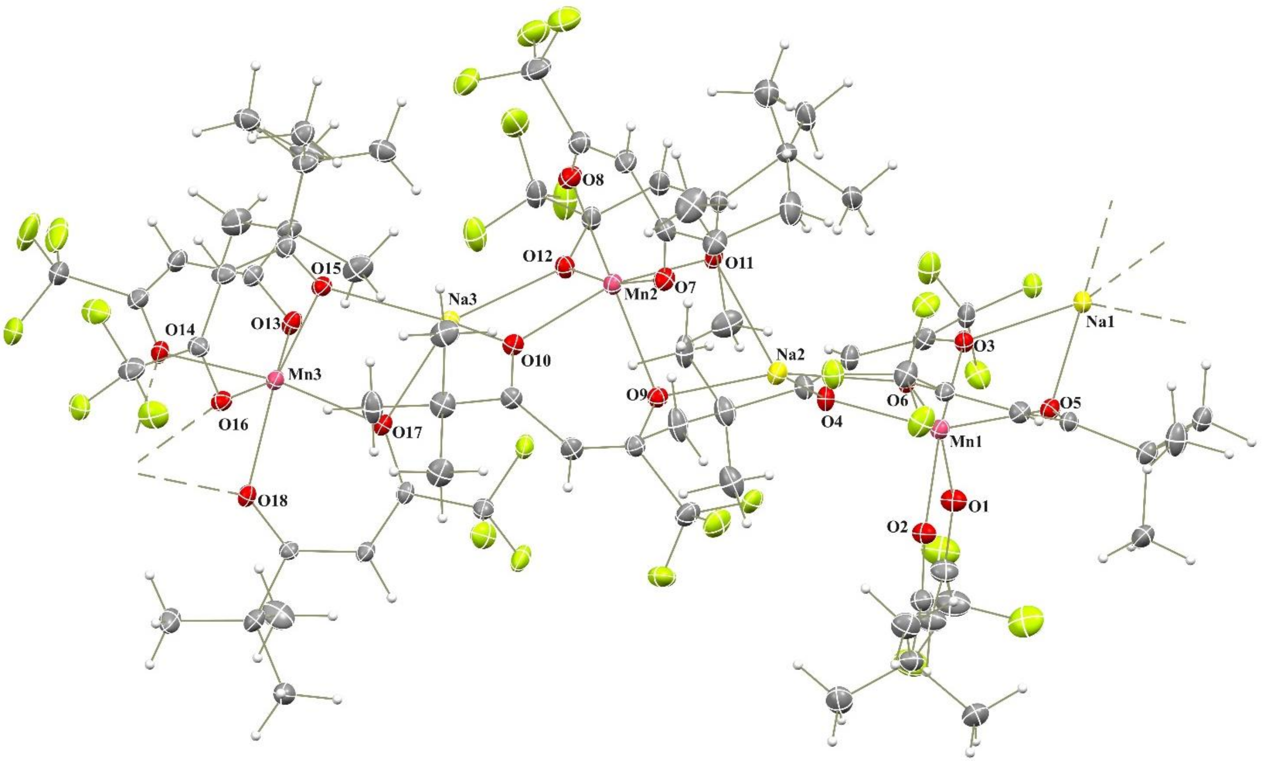

Figure S9. Solid-state structure of [NaMn(ptac)$_3$] drawn with thermal ellipsoids at the 30% probability level. Hydrogen atoms are represented by spheres of arbitrary radius.

Table S12. Bond Distances (Å) and Angles (deg.) in the Structure of [NaMn(ptac)$_3$].

**Bond distances**					
Mn(1)-O(1)	2.122(6)	Mn(2)-O(7)	2.087(5)	Mn(3)-O(13)	2.108(5)
Mn(1)-O(2)	2.129(6)	Mn(2)-O(8)	2.126(7)	Mn(3)-O(14)	2.154(5)
Mn(1)-O(3)	2.171(5)	Mn(2)-O(9)	2.179(5)	Mn(3)-O(15)	2.176(6)
Mn(1)-O(4)	2.171(5)	Mn(2)-O(10)	2.133(6)	Mn(3)-O(16)	2.183(5)
Mn(1)-O(5)	2.148(5)	Mn(2)-O(11)	2.163(5)	Mn(3)-O(17)	2.124(5)
Mn(1)-O(6)	2.172(5)	Mn(2)-O(12)	2.166(5)	Mn(3)-O(18)	2.179(6)
Na(1)-O(3)	2.312(6)	Na(2)-O(4)	2.401(6)	Na(3)-O(10)	2.402(6)
Na(1)-O(5)	2.574(6)	Na(2)-O(6)	2.347(6)	Na(3)-O(12)	2.304(6)
Na(1)-O(14)	2.419(6)	Na(2)-O(9)	2.359(6)	Na(3)-O(15)	2.380(6)
Na(1)-O(16)	2.499(6)	Na(2)-O(11)	2.350(6)	Na(3)-O(17)	2.316(6)
Na(1)-O(18)	2.468(5)				

Angles					
O(1)-Mn(1)-O(2)	82.3(2)	O(7)-Mn(2)-O(8)	83.6(2)	O(13)-Mn(3)-O(14)	83.1(2)
O(1)-Mn(1)-O(3)	83.4(2)	O(7)-Mn(2)-O(9)	86.4(2)	O(13)-Mn(3)-O(15)	91.0(2)
O(1)-Mn(1)-O(4)	94.8(2)	O(7)-Mn(2)-O(10)	101.3(2)	O(13)-Mn(3)-O(16)	155.1(2)
O(1)-Mn(1)-O(5)	106.0(2)	O(7)-Mn(2)-O(11)	94.3(2)	O(13)-Mn(3)-O(17)	101.4(2)
O(1)-Mn(1)-O(6)	164.3(2)	O(7)-Mn(2)-O(12)	169.2(2)	O(13)-Mn(3)-O(18)	108.0(2)
O(2)-Mn(1)-O(3)	165.5(2)	O(8)-Mn(2)-O(9)	168.8(2)	O(14)-Mn(3)-O(15)	106.1(2)
O(2)-Mn(1)-O(4)	101.8(2)	O(8)-Mn(2)-O(10)	95.5(2)	O(14)-Mn(3)-O(16)	76.5(2)
O(2)-Mn(1)-O(5)	95.4(2)	O(8)-Mn(2)-O(11)	103.8(2)	O(14)-Mn(3)-O(18)	87.6(2)
O(2)-Mn(1)-O(6)	83.6(2)	O(8)-Mn(2)-O(12)	88.2(2)	O(15)-Mn(3)-O(16)	81.3(2)
O(3)-Mn(1)-O(4)	81.7(2)	O(10)-Mn(2)-O(9)	81.7(2)	O(15)-Mn(3)-O(18)	157.98(19)
O(3)-Mn(1)-O(6)	110.8(2)	O(10)-Mn(2)-O(11)	156.4(2)	O(17)-Mn(3)-O(14)	170.9(2)
O(4)-Mn(1)-O(6)	81.29(19)	O(10)-Mn(2)-O(12)	86.4(2)	O(17)-Mn(3)-O(15)	81.8(2)
O(5)-Mn(1)-O(3)	86.3(2)	O(11)-Mn(2)-O(9)	81.8(2)	O(17)-Mn(3)-O(16)	100.9(2)
O(5)-Mn(1)-O(4)	154.6(2)	O(11)-Mn(2)-O(12)	80.9(2)	O(17)-Mn(3)-O(18)	83.5(2)
O(5)-Mn(1)-O(6)	82.23(19)	O(12)-Mn(2)-O(9)	102.3(2)	O(18)-Mn(3)-O(16)	85.43(19)
O(3)-Na(1)-O(5)	74.17(18)	O(6)-Na(2)-O(4)	73.13(19)	O(12)-Na(3)-O(10)	77.4(2)
O(3)-Na(1)-O(14)	113.6(2)	O(6)-Na(2)-O(9)	148.9(2)	O(12)-Na(3)-O(15)	138.9(2)
O(3)-Na(1)-O(16)	156.9(2)	O(6)-Na(2)-O(11)	122.8(2)	O(12)-Na(3)-O(17)	138.8(2)
O(3)-Na(1)-O(18)	129.9(2)	O(9)-Na(2)-O(4)	129.6(2)	O(15)-Na(3)-O(10)	118.6(2)
O(14)-Na(1)-O(5)	165.0(2)	O(11)-Na(2)-O(4)	108.7(2)	O(17)-Na(3)-O(10)	112.0(2)
O(14)-Na(1)-O(16)	66.17(19)	O(11)-Na(2)-O(9)	74.3(2)	O(17)-Na(3)-O(15)	73.68(19)
O(14)-Na(1)-O(18)	75.72(19)				
O(16)-Na(1)-O(5)	101.5(2)				
O(18)-Na(1)-O(5)	109.89(19)				
O(18)-Na(1)-O(16)	73.15(19)				

11. References

[1] SAINT; part of Bruker APEX3 software package (version 2017.3-0): Bruker AXS, **2017**.

[2] SADABS; part of Bruker APEX3 software package (version 2017.3-0): Bruker AXS, **2017**.

[3] G. M. Sheldrick, Crystal structure solution with ShelXT, *Acta Crystallogr.* **2015**, *A71*, 3-8.

[4] G. M. Sheldrick, Crystal structure refinement with SHELXL, *Acta Crystallogr.* **2015**, *C71*, 3-8.

[5] O. V. Dolomanov, L. J. Bourhis, R. J. Gildea, J. A. K. Howard, H. Puschmann, OLEX2: a complete structure solution, refinement and analysis program, *J. Appl. Crystallogr.* **2009**, *42*, 339-341.

[6] R. Wulf, Experimental distinction of elements with similar atomic number using anomalous dispersion (δ synthesis): an application of synchrotron radiation in crystal structure analysis, *Acta Crystallogr.* **1990**, *A46*, 681-688.

[7] Y. Waseda, Anomalous X-Ray Scattering for Material Characterization. Springer-Verlag Berlin, Heidelberg, **2002**.

[8] S. J. Tereniak, R. K. Carlson, L. J. Clouston, V. G. Young, E. Bill, R. Maurice, Y. S. Chen, H. J. Kim, L. Gagliardi, C. C. Lu, Role of the metal in the bonding and properties of bimetallic complexes involving manganese, iron, and cobalt, *J. Am. Chem. Soc.* **2014**, *136*, 1842-1855.

[9] D. L. Miller, R. B. Siedschlag, L. J. Clouston, V. G. Young, Y. S. Chen, E. Bill, L. Gagliardi, C. C. Lu, Redox Pairs of Diiron and Iron–Cobalt Complexes with High-Spin Ground States, *Inorg. Chem.* **2016**, *55*, 9725-9735.

[10] D. T. Cromer, D. A. Liberman, Anomalous dispersion calculations near to and on the long-wavelength side of an absorption edge, *Acta Crystallogr.* **1981**, *A37*, 267-268.

[11] Rigaku Corporation. Rigaku Oxford Diffraction, CrysAlisPro Software System, Version 1.171.43.124a. **2024**.

[12] Rigaku Oxford Diffraction. SCALE3 ABSPACK; A Rigaku Oxford Diffraction Program (1.0.11,Gui:1.0.7) (C). **2005**.
